# Recent Advances in Visible-Light-Mediated Amide Synthesis

**DOI:** 10.3390/molecules27020517

**Published:** 2022-01-14

**Authors:** Bin Lu, Wen-Jing Xiao, Jia-Rong Chen

**Affiliations:** 1 Key Laboratory of Pesticides and Chemical Biology of the Ministry of Education, College of Chemistry, Central China Normal University, 152 Luoyu Road, Wuhan 430079, China; ccnulubin@163.com (B.L.); wxiao@mail.ccnu.edu.cn (W.-J.X.); 2 School of Chemistry and Chemical Engineering, Henan Normal University, 46 East of Construction Road, Xinxiang 453007, China

**Keywords:** visible light, photoredox catalysis, photochemistry, radical chemistry, amide synthesis, amides

## Abstract

Visible-light photoredox catalysis has attracted tremendous interest within the synthetic community. As such, the activation mode potentially provides a more sustainable and efficient platform for the activation of organic molecules, enabling the invention of many controlled radical-involved reactions under mild conditions. In this context, amide synthesis via the strategy of photoredox catalysis has received growing interest due to the ubiquitous presence of this structural motif in numerous natural products, pharmaceuticals and functionalized materials. Employing this strategy, a wide variety of amides can be prepared effectively from halides, arenes and even alkanes under irradiation of visible light. These methods provide a robust alternative to well-established strategies for amide synthesis that involve condensation between a carboxylic acid and amine mediated by a stoichiometric activating agent. In this review, the representative progresses made on the synthesis of amides through visible light-mediated radical reactions are summarized.

## 1. Introduction

### 1.1. General Strategies for Amide Bond Formation

**Figure 1:**
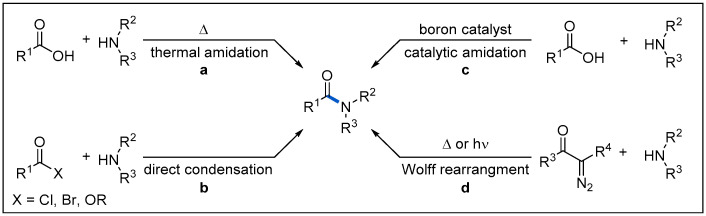
Selected traditional methods for amide synthesis.

The amide motif is the backbone of peptides and an important structural unit for many natural products and functional materials [1,2,3,4,5]. According to the statistics in a 2006 survey, amides are found in two-thirds of drug candidates [6] and present in 25% of all pharmaceuticals currently in the market [7]. In addition, in 2014, more than 50% of the reported processes used amidation reactions [8]. There is no doubt that the synthesis of amides is one of the most fundamental reactions in medicinal chemistry. Methodologies for the synthesis of amides have also emerged, mainly including four categories (Figure 1) [9].

The most direct and ideal method for amide synthesis involves dehydration between carboxylic acids and amines (Figure 1, a), but this process usually requires an elevated temperature. Direct coupling of activated carboxylic acid derivatives and amines in the presence of external activating reagents represents the most classic method for amide synthesis [10,11] (Figure 1, b). This method, however, requires the initial or in situ conversion of the carboxylic acid to the corresponding more active acid halides, mixed anhydrides, or activated esters in the presence of an external activating reagent. It has the characteristics of mild reaction conditions, high efficiency and is especially popular in laboratory synthesis. In addition, the catalytic amidation mediated by boron and element of group IV has also witnessed considerable progress [12,13]; however, the main drawback is that it tends to lack catalytic activity in the cases of more challenging substrates (Figure 1, c). Moreover, thermal or photoinitiated Wolff rearrangement of α-diazol ketones to ketenes, followed by the nucleophilic addition of amines constitutes a powerful way to prepare amides. (Figure 1, d) [14,15,16,17]. The obvious advantage of this method is that the substrates are not limited to common carboxylic acids. It can be extended to alkyl ketones so that it can provide an effective idea for more complex molecular synthesis. These synthetic methods have been well explored and comprehensively reviewed [8,9,10,11,12,13,14,15,16,17]. With the evolvement of visible-light-driven photoredox catalysis, amide synthesis via the strategy of photoredox catalysis has received growing interest. In this review, we summarize recent examples of the construction of amides through visible-light-mediated radical reactions. Specifically, we first introduce the basic activation modes of visible-light photoredox catalysis. Then, we describe the recent representative works in the field of visible-light-mediated amide synthesis according to different reaction types and radical precursors. Finally, we summarize the progress in this field and propose an outlook.

### 1.2. Visible-Light Photoredox Catalysis

**Figure 2:**
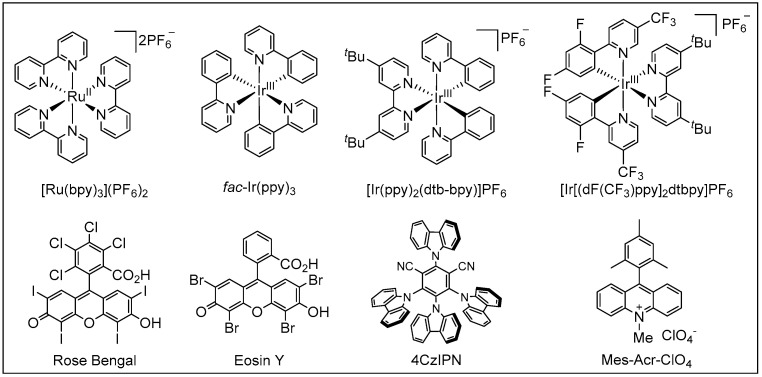
Typical metal-based or organic photoredox catalysts.

**Figure 3:**
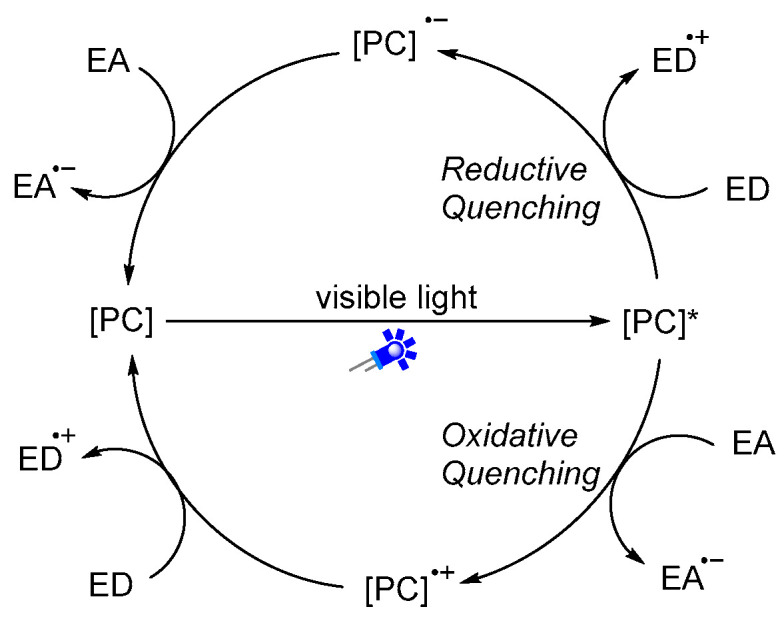
General mechanism of visible-light-driven photoredox catalysis.

Visible light is an inexhaustible energy source with environmentally friendly characteristics. Unlike traditional ionic reactions, visible-light-mediated radical reactions can usually break and recombine chemical bonds under milder and more sustainable conditions, which provides new platforms for the formation of chemical bonds in a controlled manner. Given these advantages, over the past years, photoredox catalysis has emerged as a valuable synthetic tool in synthetic organic chemistry [18,19,20,21,22,23,24,25,26,27,28,29]. Photoredox catalysis usually requires the addition of metal-based complexes or organic dyes as photosensitizers (Figure 2), which can then activate substrates into radical species through the single-electron transfer (SET) process. A general mechanism of photoredox catalysis is shown in Figure 3. An excited state photocatalyst [PC]* was initially generated by irradiation of the photocatalyst [PC], which has an altered electronic distribution caused by the metal-to-ligand charge transfer (MLCT). Then, this excited state photocatalyst [PC]*, which can be considered as a more potent reductant or stronger oxidant, reacts with electron-deficient substrate, an electron acceptor [ED], or electron-rich substrate, an electron donor [EA], by donating or accepting an electron via a SET process. Specifically, the oxidized [PC]^•+^ or reduced [PC]^•−^ can then undergo a SET process with either the substrate or other intermediate to regenerate the ground state catalyst. Recently, photoredox catalysis has also found wide applications in the synthesis of amides. In this review, we will summarize the recent representative examples of the construction of amides through visible-light-mediated radical reactions. We hope it will be useful for synthetic organic chemists and will inspire further reaction development in this area.

## 2. Oxidative Amidations

### 2.1. From Keto Acids

**Figure 4:**
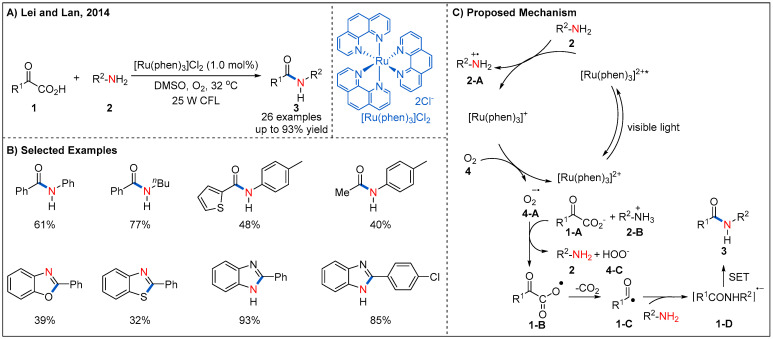
Visible-light-mediated decarboxylation/oxidative amidation of α-keto acids.

Initial efforts in applying visible-light photoredox catalysis for the radical oxidative decarboxylative coupling of α-keto acids **1** with amines **2** were described by Lei and Lan (Figure 4) [30]. The reaction proceeds smoothly at room temperature with [Ru(phen)_3_]Cl_2_ as a photocatalyst and a commercial household fluorescent lamp as a light source. A range of differently substituted aromatic α-keto acids **1** undergo a decarboxylative amination efficiently, providing amide products **3** in generally good yields. Alkyl α-keto acids can also react with 4-methylaniline, delivering desired products in moderate yields. Interestingly, anilines containing NH_2_, OH, and SH groups at ortho-position react with α-keto acids smoothly to afford heterocyclic compounds in moderate to good yields. This protocol provides a novel and straightforward method for the synthesis of amides and heterocyclic compounds under very mild reaction conditions. The authors proposed a reasonable catalytic cycle (Figure 4C). Irradiation of the ground state [Ru(phen)_3_]^2+^ with visible-light leads to the excited state [Ru(phen)_3_]^2+*^ by metal-to-ligand charge transfer (MLCT). Then, electron transfer occurs directly between the [Ru(phen)_3_]^2+*^ and amines **2** to form nitrogen radical cation **2-A** and reduced state [Ru(phen)_3_]^+^. Then, O_2_ oxidizes [Ru(phen)_3_]^+^ back to[Ru(phen)_3_]^2+^ by a SET process and generates the superoxide radical anion **4-A**. This superoxide radical anion then undergoes a SET event with α-keto acid anion **1-A** to generate carboxylic oxygen radical **1-B** and peroxyacid anion **4-C**. The carboxylic oxygen radical **1-B** subsequent undergoes a decarboxylation to form acyl radical **1-C**, which may subsequently react with an amine to deliver the amide radical anion **1-D**. Finally, **1-D** undergoes another SET process to give the desired product.

### 2.2. From Alcohols

**Figure 5:**
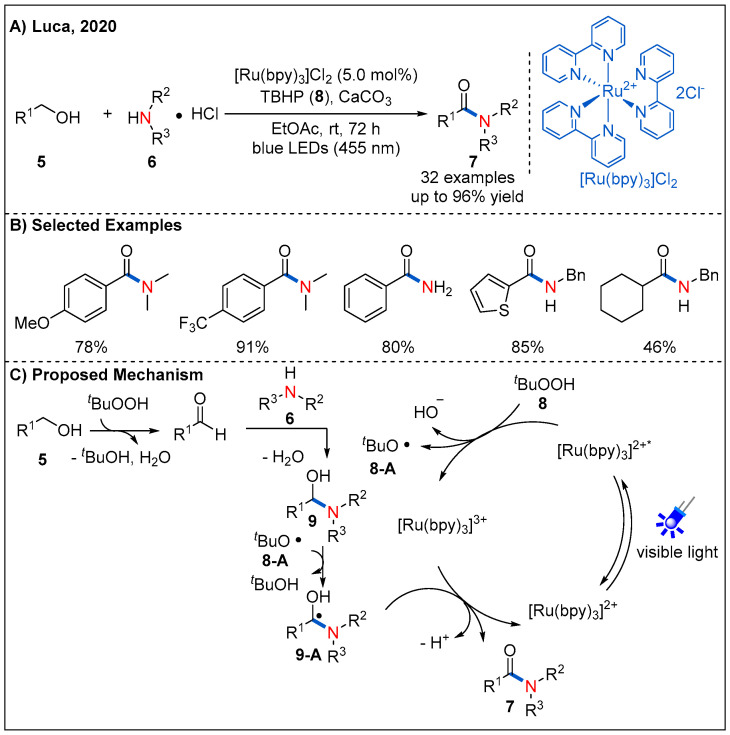
Oxidative amidation of x’alcohols.

Alcohols are easily available feedstock and such functionality is commonly found in many natural products. Direct conversion of alcohols to amides is a focus of sustainable chemistry. In 2020, Luca’s group reported a photocatalyzed route to amides from alcohols **5** and amines **6** (Figure 5) [31]. As shown in Figure 5C, the proposed catalytic cycle starts with a SET event from the excited state of [Ru(bpy)_3_]^2+^* to *tert*-butyl hydroperoxide (TBHP), forming the *tert*-butoxy radical **8-A** and hydroxyl anion. Next, tert-butoxy radical **8-A** abstracts a hydrogen atom from the in situ-formed hemiaminal **9** to form intermediate **9-A**. Then, **9-A** undergoes deprotonation to give the ketyl radical anion, which then reduces the Ru(III) complex by a SET event to form amide product **7**. At the same time, photoredox catalytic cycle is completed, with the regeneration of the ground state photocatalyst. Later, a similar method was reported by Panda and Ghosh using Cu-N-TiO_2_ heterogeneous photocatalyst [32].

### 2.3. From Amines

**Figure 6:**
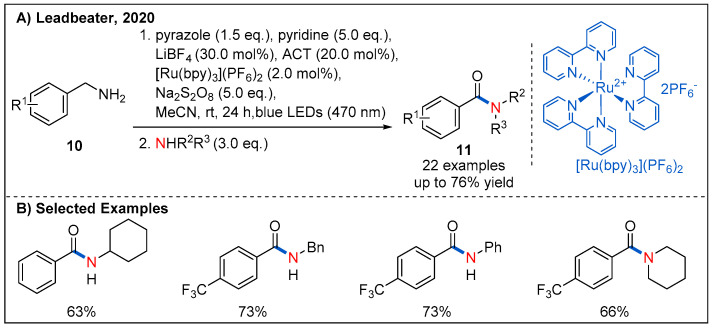
Oxidative amidation of amines and transamidation.

In 2020, Leadbeater’s group reported an interesting route for the synthesis of amides by visible-light-promoted oxidative amidation and transamidation (Figure 6). In this process, sodium persulfate was employed as a terminal oxidant, and benzylamines with different substituents could be converted to the corresponding amides **11** in generally good yields [33].

### 2.4. From Aldehydes

**Figure 7:**
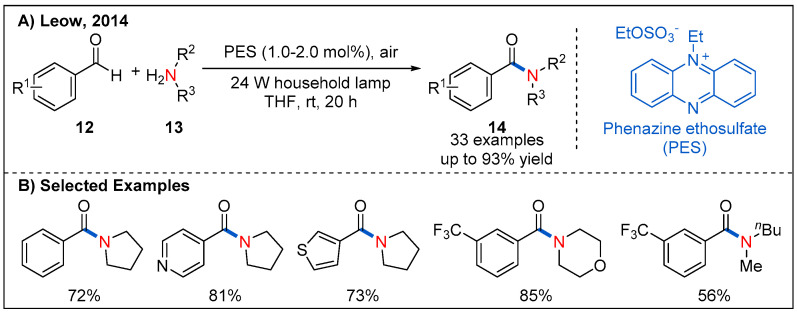
Oxidative amidation and transamidation between aldehydes and amines.

**Figure 8:**
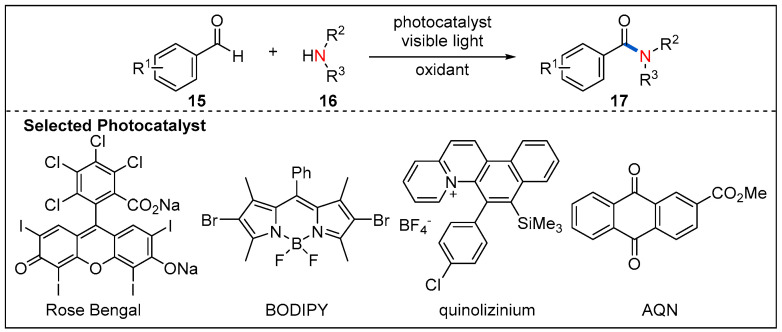
Selected organic photocatalysts for oxidative amidation of aldehydes.

Photocatalytic oxidative coupling of aldehydes and amines using molecular oxygen rather than stoichiometric additive as oxidants represents a green and alternative route to amides. In 2014, the oxidative amidation of aromatic aldehydes with amines is reported by Leow, which used an inexpensive phenazine ethosulfate as organic photocatalyst and air as the sole oxidant (Figure 7) [34]. A set of aromatic aldehydes undergo oxidative amidation with good yields. It is also highly desirable to search for metal-free photocatalysts with a low cost and high efficiency for the oxidative amidation of aromatic aldehydes. Recently, a similar strategy was further developed by merging different organic photocatalysts such as Rose Bengal, BODIPY, quinolizinium and AQN with additional oxidants (Figure 8) [35,36,37].

### 2.5. From Acids

**Figure 9:**
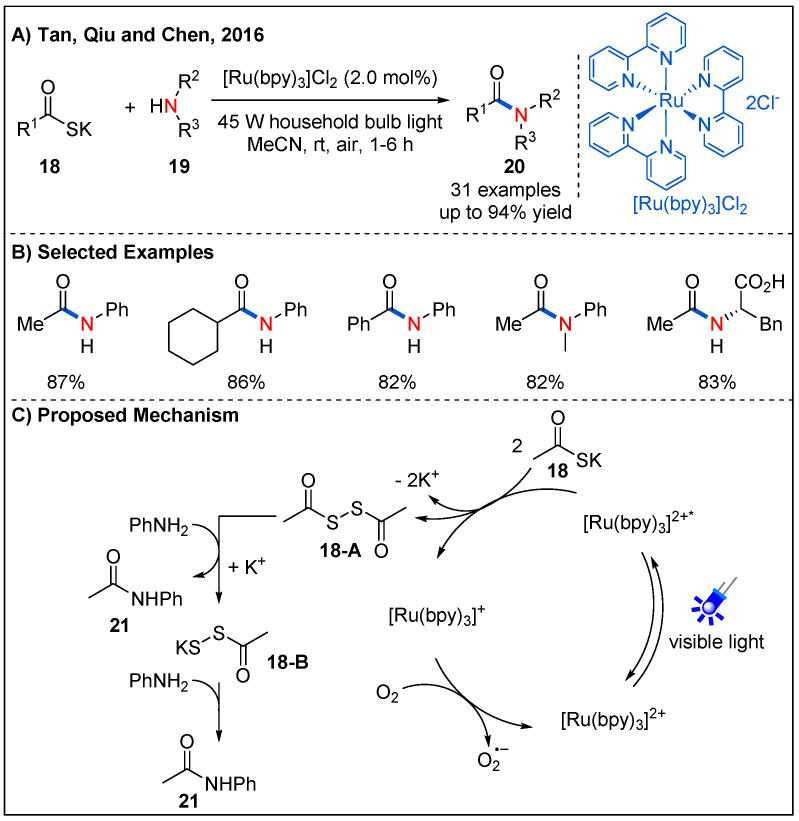
Selective amide constructions from potassium thioacids.

Although the method of construction of amides through the condensation of carboxylic acids and amines under the condition of external activating reagents has been well established, stoichiometric coupling agents decrease the atom economy. Therefore, the development of efficient methods for catalytic activation of acids is urgently needed. In 2016, Tan, Qiu and Chen reported an elegant oxidative amidation of potassium thioacids **18** with amines **19** by photoredox catalysis, providing a green catalytic process for amide formation (Figure 9) [38]. As shown in Figure 9C, preliminary mechanistic studies revealed that diacyl disulfide **18-A** derived from potassium thioacids under visible-light irradiation might be the key intermediate, and O_2_ served as a green oxidant. This method has the characteristics of mild reaction conditions and good functional group compatibility. Primary, secondary aromatic amines and even amino acids could participate in the reaction well, providing the final products in high yields (Figure 9B).

**Figure 10:**
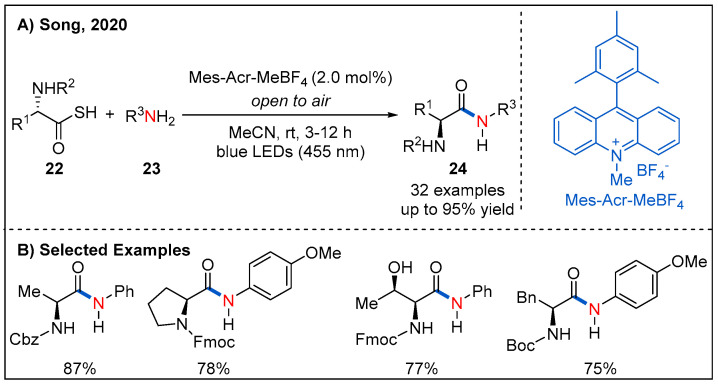
Visible light-induced amide bond formation from thioacids.

**Figure 11:**
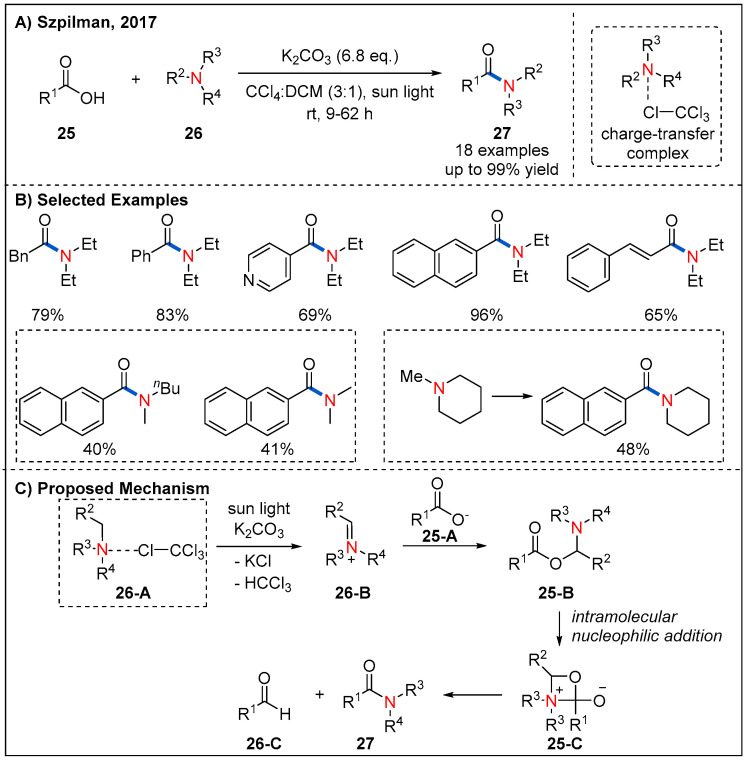
Sunlight-assisted direct dealkylative amide formation via a charge-transfer complex.

Inspired by this work, Song and co-workers reported an oxidative amidation of thioacids **22** using organic dye as photocatalyst and air as oxidant under mild reaction conditions (Figure 10) [39]. A range of amino acids were tolerated, providing chiral α-aminoamides **24** in good yields. Moreover, a visible-light-assisted direct amide formation from carboxylic acids **25** and amines **26** was developed by Szpilman et al. (Figure 11) [40]. They disclosed that trimethylamine and tetrachloromethane give rise to a light-absorbing charge-transfer complex. Irradiation of this charge-transfer complex using visible light led to the electron transfer between trimethylamine (electron donor) and tetrachloromethane (electron acceptor), thus providing iminium ion and chloroform. Based on this concept, a sunlight-assisted amidation of carboxylic acids and amines was successfully achieved by using tetrachloromethane as an electron acceptor. A variety of amines **26** underwent a dealkylative process with aliphatic or aromatic acids to generate amides **27** in moderate to good yields. Unsymmetrically substituted cyclic alkyl amine leads to amide formation with moderate yield and good chemoselectivity while dimethyl-butylamine leads to formation of a 1:1 mixture of demethylated and debutylated products in a combined 81% yield. The authors rationalized that the selectivity for the dealkylative process mainly depends on the stability of iminium ions; specifically, iminium ions with more substituents are more stable and easier to form the final amide products. The mechanism is shown in Figure 11C; irradiation of the charge-transfer complex **26-A** using sunlight leads to the formation of iminium ion **26-B**, which is then attacked by the carboxylate **25-A** to form **25-B**. Next, **25-B** undergoes an intramolecular nucleophilic addition to generate **25-C**. Finally, intermediate **25-C** collapses to form the final product **27** with the release of one equivalent of aldehyde **26-C**.

### 2.6. From Alkynes

**Figure 12:**
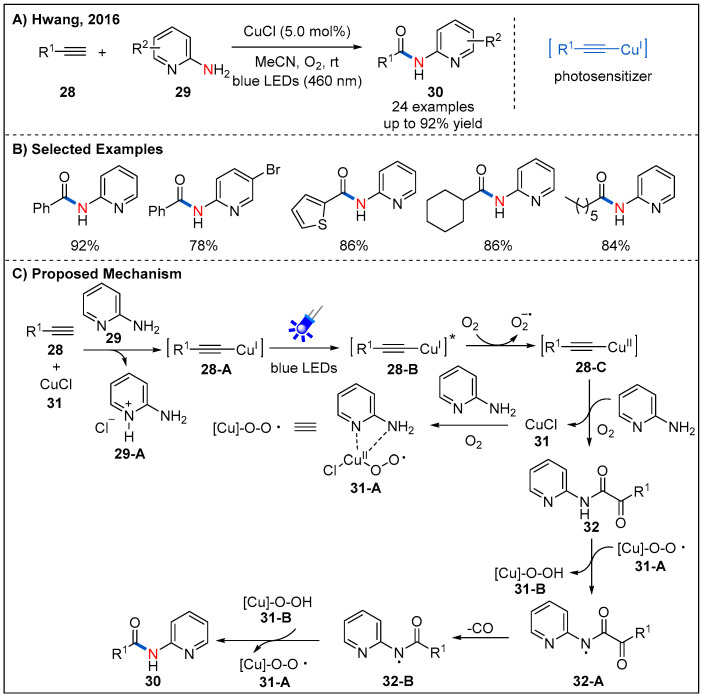
Visible-light-mediated oxidative C–N coupling.

Reactions involving C≡C triple bond cleavage is of great challenge in the construction of targeted molecules in organic synthesis. In 2016, Hwang’s group described a novel and effective copper-catalyzed aerobic oxidative C–N coupling via C≡C triple bond cleavage under visible light irradiation was (Figure 12) [41]. A wide range of substrates, including various terminal alkynes **28** and electron-deficient 2-aminopyridines **29** were well-tolerated (Figure 12B). The mechanistic investigation revealed that the copper(II)-superoxo or -peroxo complex is probably responsible for the oxidative cleavage of C≡C triple bonds of terminal alkynes. Based on a series of mechanistic experiments, a plausible mechanism was proposed (Figure 12C). In situ-generated copper(I) phenylacetylide **28-A** reaches excited state via ligand to metal charge transfer (LMCT) under irradiation of blue LEDs. Then, the photo-excited Cu(I)-phenylacetylide **28-B** undergoes a SET process with O_2_ to generate the Cu(II)-phenylacetylide **28-C** and superoxide radical anion. Nucleophilic addition of 2-aminopyridine **29** to Cu(II) phenylacetylide **28-C** in the presence of molecular oxygen resulted in the formation of complex Cu(III) species. Subsequent reductive elimination of Cu(III) to Cu(I) and reaction with molecular oxygen afford pyridine ketoamides **32**. Cu(II) superoxo/-peroxo complex **31-A** abstracts an acidic hydrogen atom to form a *N*-centred radical **32-A**. Finally, radical-assisted carbon monoxide elimination and recombination of *N*-centered radical and carbon-centered radical leads to the formation of **32-B**, which could be converted into the desired amide after abstracting a. hydrogen atom from **31-B**.

**Figure 13:**
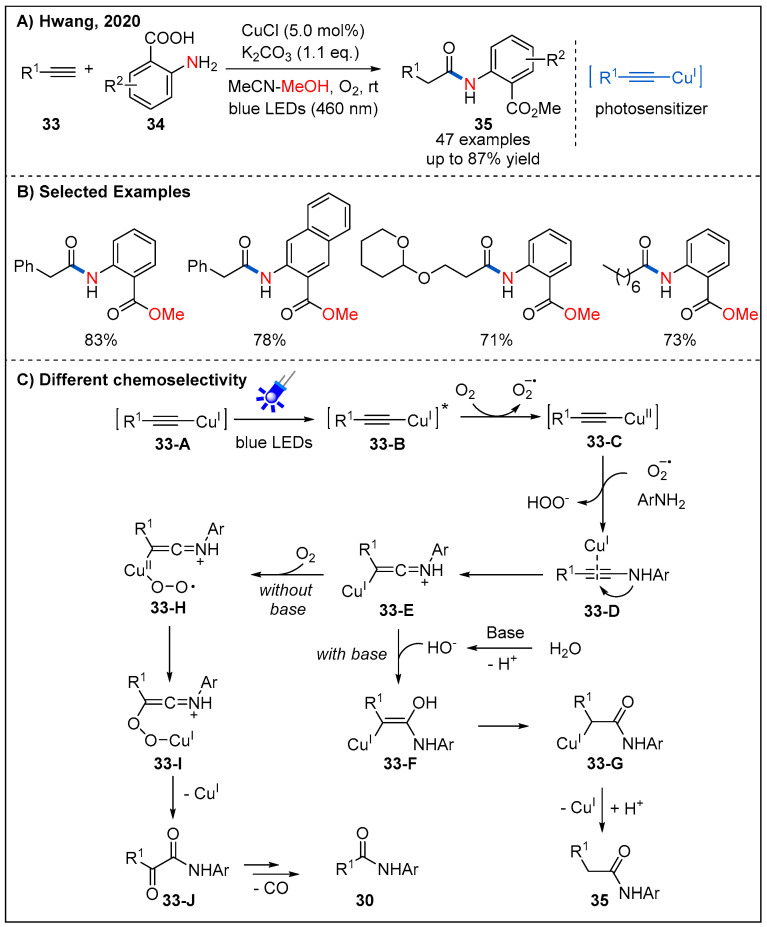
Visible light-promoted copper-catalyzed regioselective acetamidation of terminal alkynes.

Interestingly, after replacing 2-aminopyridines with aryl amines and adding an equivalent amount of potassium carbonate as an additive, the process of breaking the C≡C bond is suppressed. In 2020, Hwang and co-workers described a visible-light-promoted copper-catalyzed regioselective acetamidation of terminal alkynes **33** with arylamines **34** (Figure 13) [42]. A wide range of arylalkynes and alkylalkynes reacted smoothly, producing the corresponding amides **35** in generally good yields. The reason of selectivity in the formation of products, **30** and **35**, can be attributed to nucleophiles and bases (Figure 13C). When the base is present in the system, the nucleophilicity of water or carboxylic acid can be increased, so **33-E** is more susceptible to nucleophilic addition, followed by protonation to form amide **35**. In contrast, in the absence of a suitable nucleophile, **33-E** reacts more readily with oxygen to form **33-H**, furnishing the α-ketoamide **33-J**. Finally, in the presence of the superoxide species **31-A**, **33-J** can be converted into dealkylative amide **30**.

### 2.7. From Enamines

**Figure 14:**
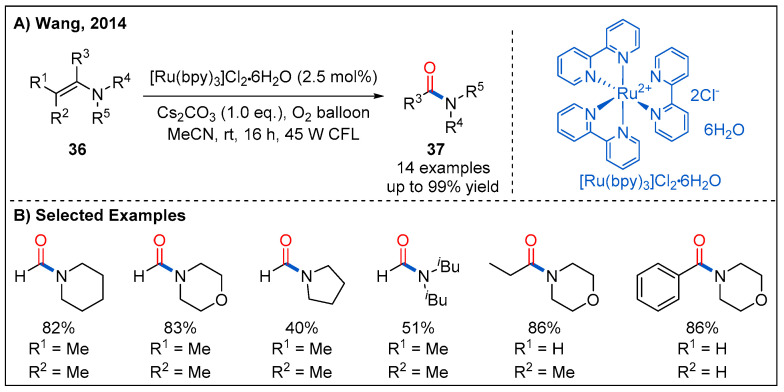
Photocatalytic aerobic oxidation of enamines to amides.

In 2014, Wang and co-workers demonstrated that enamines **36** could be smoothly converted into the amides **37** under visible-light photocatalytic conditions (Figure 14) [43]. The oxidative amidation of enamines is performed using 2.5 mol% of Ru(bpy)_3_Cl_2_·6H_2_O as photocatalyst and molecular oxygen as oxidant under irradiation of 45 W household light bulb. Amides were obtained in moderate to good yields when *N,N*-dialkyl substituted enamines were employed (Figure 14B).

## 3. Ritter-Type Reactions

**Figure 15:**
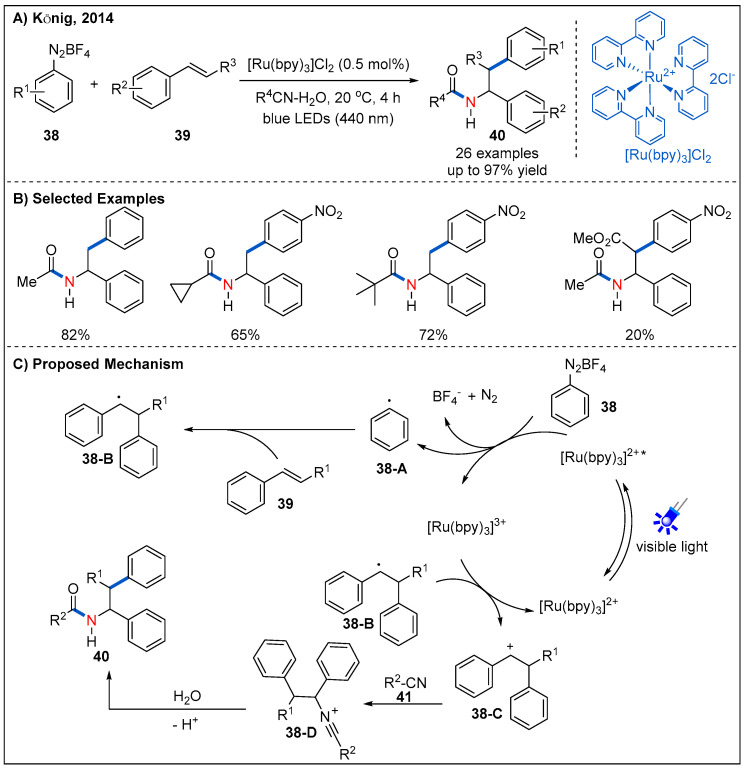
Photoredox-catalyzed Meerwein addition and Ritter-type amination reaction.

The Ritter-type amination reaction is one of the most classic and useful transformations for the formation of C-N bonds [44]. In 2014, a novel visible-light-driven aryl radical-mediated Meerwein addition reaction, namely intermolecular amino-arylation of alkenes **39**, was firstly reported by König’s group (Figure 15) [45]. They demonstrated that aryl radicals could be produced from aryl diazonium salts **38** using [Ru(bpy)_3_]Cl_2_ as a photocatalyst under irradiation of blue LEDs. This strategy affords a mild approach for intermolecular three-component amino-arylation of alkenes. The reaction tolerates a wide range of substituents on the aryl diazonium salts **38**, including -NO_2_, -OMe, -Br. Furthermore, mono-substituted, di-substituted and tri-substituted nitriles **41** were proved to be compatible with the reaction. A plausible mechanism was also proposed (Figure 15C). Firstly, aryl radical **38-A** was formed by a SET process from the excited state of the photocatalyst [Ru(bpy)_3_]^2+*^ to diazonium salt. Then, the addition of an aryl radical to alkene yielded the corresponding carbon radical intermediate **38-B**, which was then further oxidized to give carbenium intermediate **38-C**; finally, the carbenium intermediate is attacked by a nitrile, followed by hydrolysis to give the final amide product **40**.

**Figure 16:**
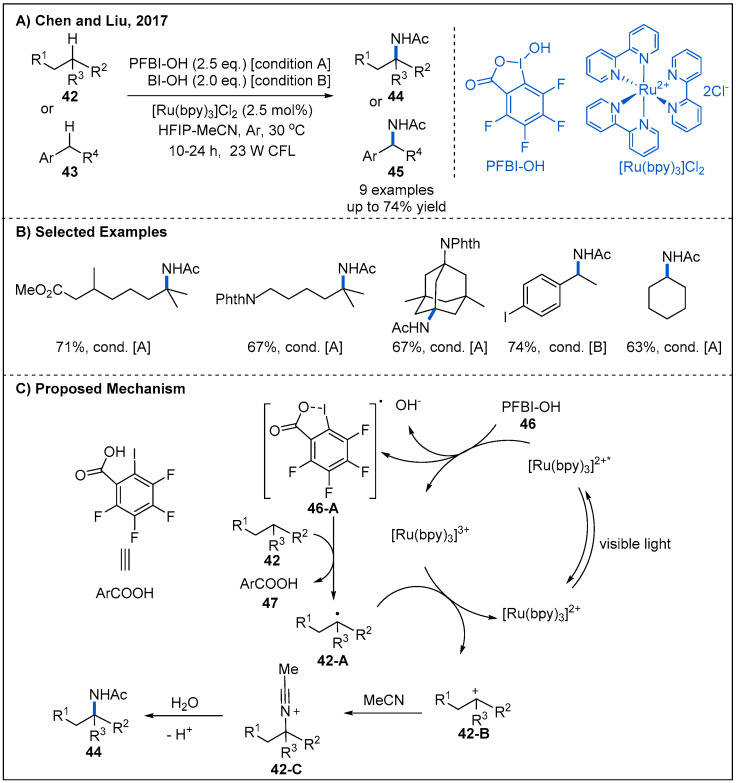
Visible-light-mediated photoredox-catalyzed oxidative C(sp^3^)–H amidation.

In 2017, Chen, Liu, and co-workers developed a photoredox catalyzed strategy for C(sp^3^)–H amidation of tertiary and benzylic C-H bonds of **42** or **43** (Figure 16) [46]. The best conditions involve using [Ru(bpy)_3_]Cl_2_ as a photoredox catalyst in the presence of hydroxyl perfluorobenziodoxole (PFBI-OH) **46** or hydroxyl benziodoxole (BI-OH) as an external oxidant. The major advantage of this catalytic system is the excellent regioselectivity of HAT process. As described in Figure 16C, the mechanism of this tertiary C–H amidation begins with a SET from the photoexcited [Ru(bpy)_3_]^2+^* to PFBl–OH, generating an oxygen radical **46-A**. This radical species abstracts a hydrogen atom from alkane, generating a tertiary carbon radical **42-A**. Tertiary carbon radical can then be oxidized by [Ru(bpy)_3_]^3+^, forming tertiary carbocation intermediate **42-B** and regenerating the photocatalyst. Finally, trapping of tertiary carbocation **42-B** by MeCN, followed by hydrolysis to yield the corresponding amide via a Ritter-type reaction mechanism.

## 4. Carbamoylation

**Figure 17:**
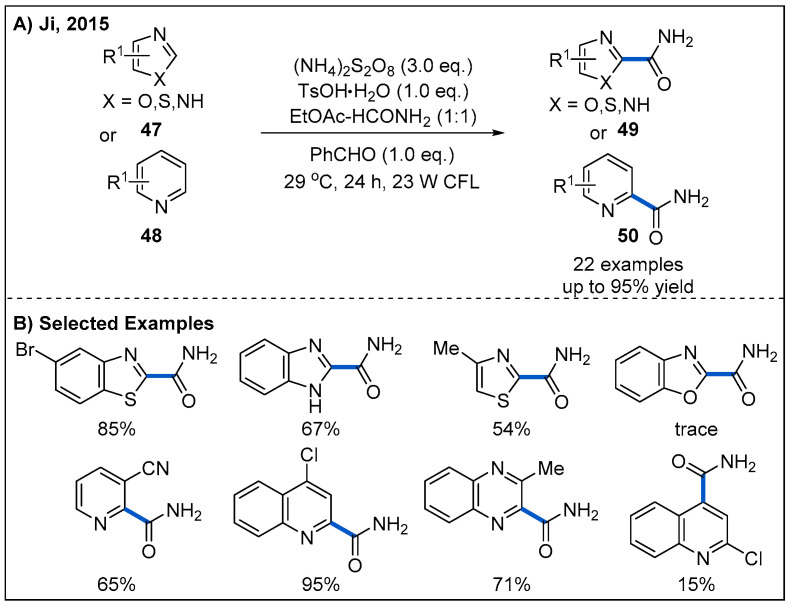
Photoredox-catalyzed cross-dehydrogenative coupling (CDC) reaction for the synthesis of amides.

Visible-light-mediated carbamoylation provides another alternative way for the synthesis of amides. In 2016, Ji and co-workers reported a photoinduced cross-dehydrogenative coupling (CDC) reaction to construct amides by reacting a variety of five- and six-membered electron-deficient heteroarenes **47** and **48** with formamide under visible-light irradiation (Figure 17) [47]. In this process, (NH_4_)_2_S_2_O_8_ was employed as an oxidant. The optimized reaction conditions were found to be using benzaldehyde as a photosensitizer and household CFL bulbs as the light source. A wide range of heteroarenes were carbamoylated in good to high yields. However, benzoxazole was not suitable for this transformation (Figure 17B).

**Figure 18:**
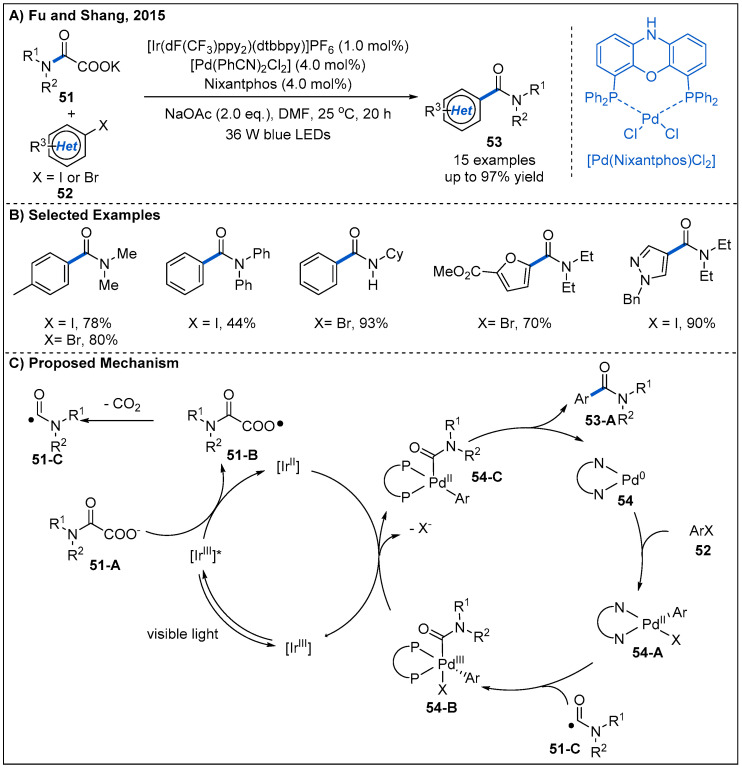
Visible-light-mediated dual photoredox and palladium-catalyzed decarboxylative couplings between α-oxocarboxylates and aryl halides.

In 2015, decarboxylative couplings of potassium oxalate monoamides **51** with aryl halides **52** by merging photoredox with palladium catalysis were reported by Fu and Shang (Figure 18) [48]. This dual catalytic system allows the formation of amides at room temperature, while direct decarboxylative coupling of potassium oxalate monoamides with aryl halides is difficult [49]. On the basis of this strategy, a diverse range of heteroaromatic amides **53** containing furan, pyrazole and pyrimidine rings can be obtained in good yields. A plausible mechanism is also proposed (Figure 18C). Initially, photoexcited [Ir(dF(CF_3_)ppy)_2_(dtbbpy)]^+^* undergoes a reductive quenching with potassium oxalate monoamide **51-A**, forming an oxygen radical **51-B** and [Ir(dF(CF_3_)ppy)_2_(dtbbpy)]^0^. The radical **51-B** then undergoes a subsequent decarboxylation to generate acyl radical **51-C**. At the same time, Pd(0) complex **54** undergoes an oxidative addition with aryl halide **52** to generate Pd(II) complex **54-A**. Then, Pd(II) complex captures the acyl radical **51-C** to generate a Pd(III) intermediate **54-B**, which oxidizes Ir(II) to Ir(III) to complete the photoredox catalytic cycle and forms a new Pd(II) intermediate **54-C**. Finally, Pd(II) intermediate **54-C** undergoes a reductive elimination to deliver the final amide product and finish the palladium catalytic cycle.

**Figure 19:**
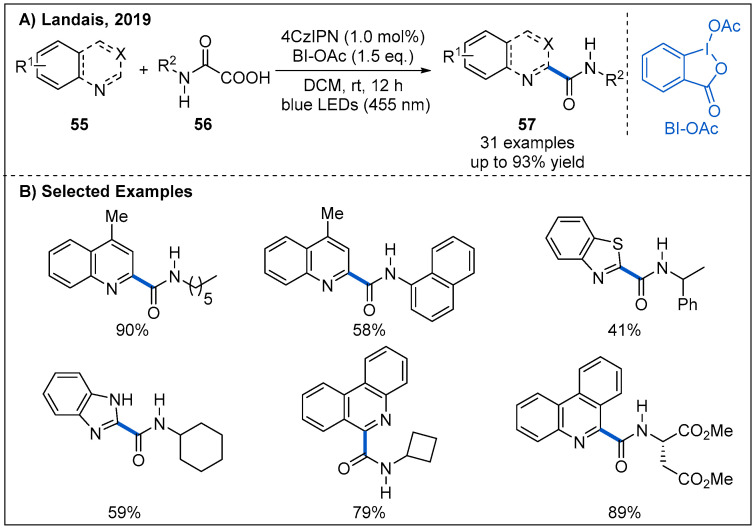
Amide synthesis by photoredox-catalyzed carbamoyl radical addition to heteroarenes.

Later, a metal-free visible-light-mediated photoredox-catalyzed carbamoyl radical addition to heteroarenes was achieved by Landais et al. (Figure 19) [50]. In this process, decarboxylative couplings of heterocycles **55** with oxamic acids **56** proceeded smoothly in the presence of 4CzIPN as an organic photocatalyst and hypervalent iodine BI-OAc as an oxidant under blue LED irradiation [51,52]. After establishing the optimal reaction condition, the generality of the transformation was subsequently evaluated. A wide range of oxamic acids and amino-acid derived oxamic acids reacted well with electron deficient heteroaryl cycles, providing the corresponding heteroaromatic amides 57 in moderate to good yields. Notably, the addition of carbamoyl radicals to quinoline has an excellent regioselectivity. Moreover, α-aminoacid-derived oxamic acids were also compatible with this catalytic system, leading to the corresponding amides without racemization (Figure 19B).

**Figure 20:**
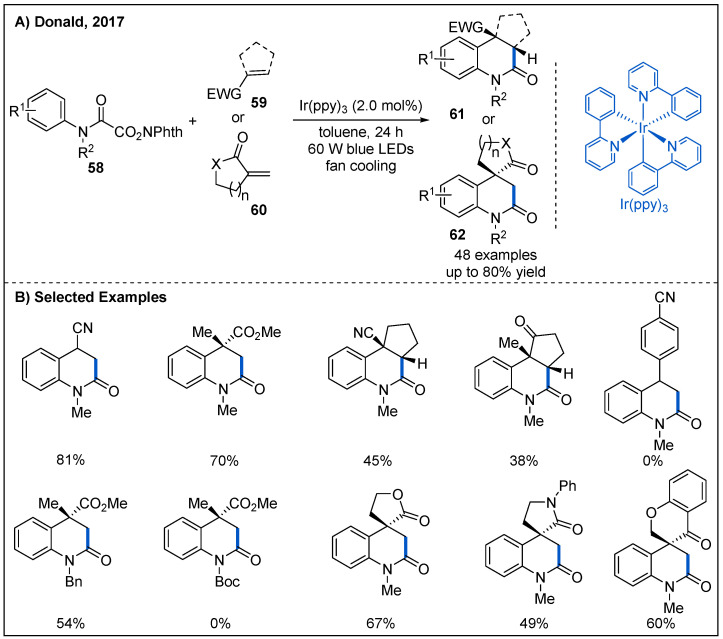
Amide synthesis by intermolecular decarboxylative addition/cyclization.

In 2017, Donald and co-workers disclosed an intermolecular decarboxylative addition/cyclization of N-hydroxyphthalimido oxamides **58** and electron-deficient alkenes **59** or **60** using Ir(ppy)_3_ as photocatalyst under blue LED irradiation (Figure 20) [53]. The reaction gave rise to valuable substituted 3,4-dihydroquinolin-2-ones in moderate to good yields. Mono-substituted, electron-deficient alkenes were well-tolerated, but substituted styrenes failed to participate in the reaction. Notably, spirocyclic cycloalkanone-lactam or lactone could be obtained in moderate yields when cyclic α,β-unsaturated esters or amides featuring an exocyclic C=C bond were used to trap the carbamoyl radical intermediates. Furthermore, the protecting group of nitrogen is important for this transformation. When the methyl group at the nitrogen atom was replaced with Boc group, the reaction was inhibited.

**Figure 21:**
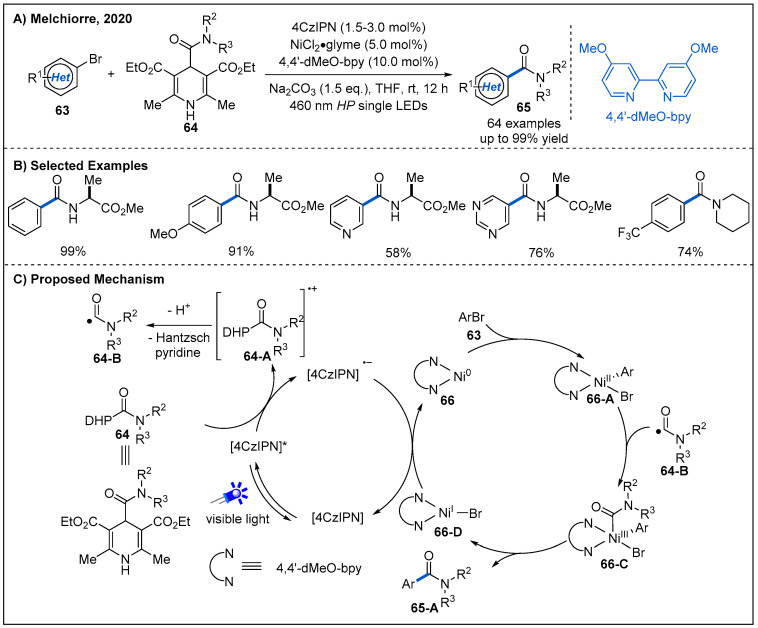
Amide synthesis by nickel/photoredox-catalyzed direct carbamoylation of (hetero)aryl bromides.

In 2020, a new class of carbamoyl radical precursors **64** derived from 4-acyl-1,4-dihydropyridines [54,55] were developed by Melchiorre’s group [56]. These radical precursors are bench-stable and can be easily prepared in a modular fashion from dihydropyridine derivatives with a carboxyl moiety at the C4-position. Employing such carbamoyl radical precursors, Melchiorre and co-workers developed a cross-coupling reaction between substituted dihydropyridines **64** and aromatic bromides **63** by merging nickel and photoredox catalysis (Figure 21). The reaction proceeded smoothly at ambient temperature and proved to be tolerant of a range of sensitive-functional-group-containing substrates. Notably, amide functionalities with electron-poor and sterically demanding amine were well accommodated. This protocol also allowed the installation of the amide scaffold within biologically relevant heterocycles (Figure 21B). On the basis of cyclic voltammetry experiments, a possible mechanism of this carbamoylation process is proposed (Figure 21C). Initially, photo-excited organic photocatalyst [4CzIPN]* oxidized dihydropyridine derivative **64** to generate a carbamoyl radical **64-B** and reductive [4CzIPN]^•−^ via a SET event. At the same time, Ni(0) complex **66** undergoes oxidative addition with aromatic bromide to form Ni(II) complex **66-A**. Then, Ni(II) complex captures the carbamoyl radical **64-B** to afford a Ni(III) intermediate **66-C**, which subsequently undergoes a reductive elimination to deliver the product and generates a Ni(I) complex **66-D**. The Ni(I) complex oxidizes reductive [4CzIPN]^•−^ to [4CzIPN], with regenerating Ni(0) complex and completing both photoredox and nickel catalytic cycles.

**Figure 22:**
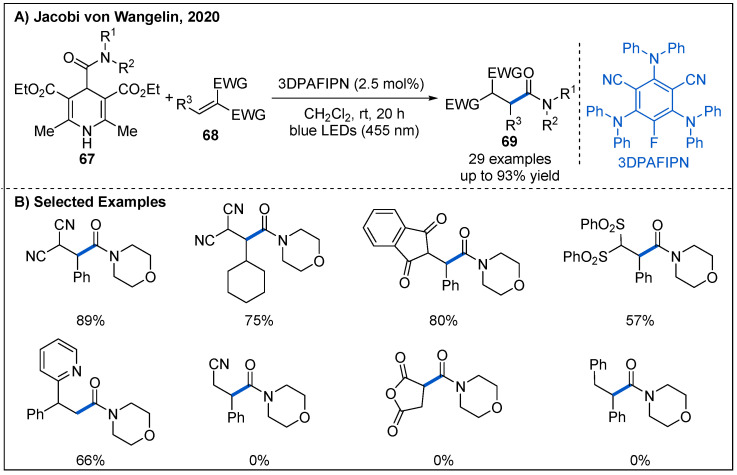
Amide synthesis by photoredox-catalyzed addition of 4-carboxamido-1,4-dihydropyridine to olefins.

At the same time, Jacobi von Wangelin’s group independently reported a practical photoredox-catalyzed addition of 4-carboxamido-Hantzsch ester-derived carbamoyl radicals to olefins under photoredox-catalyzed conditions (Figure 22) [57]. The catalytic system involves using 2.5 mol% 3DPAFIPN as organic photocatalyst under irradiation of blue LEDs. In this process, 1,1-disubstituted alkenes with an electron-deficient group such as CN and SO_2_Ph are all well-tolerated, affording the corresponding amides with high levels of regio- and chemoselectivity in generally good yields. In addition, 1,1-diarylalkenes are also good partners for the reaction. However, stilbene and maleic anhydride were not suitable for this reaction.

**Figure 23:**
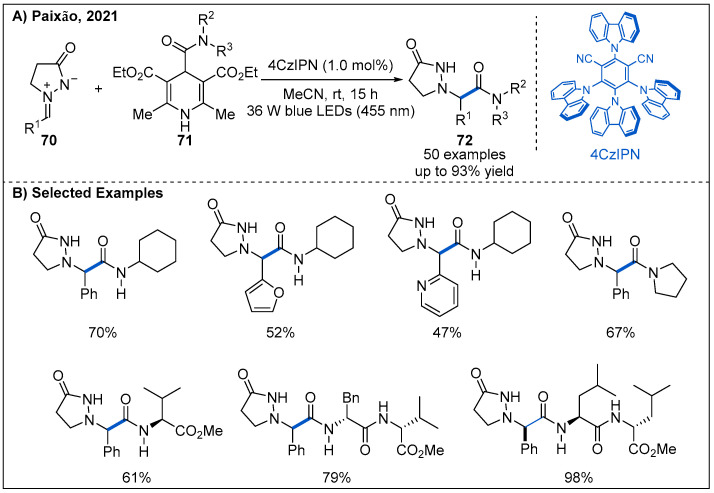
Visible-light-driven photoredox-catalyzed carbamoylation of azomethine imines.

Recently, photocatalytic methodology to install the amide functional group into azomethine imine ions is described by Paixão (Figure 23) [58]. The addition reaction of 4-carbamoyl-1,4-dihydropyridine-derived carbamoyl radicals to azomethine imines **70** allows construction of a set of *β*-alanine analogues. Aryl azomethine imines bearing either electron-withdrawing or electron-donating substituents all undergo carbamoylation efficiently, affording the amide products in moderate to good yields.

**Figure 24:**
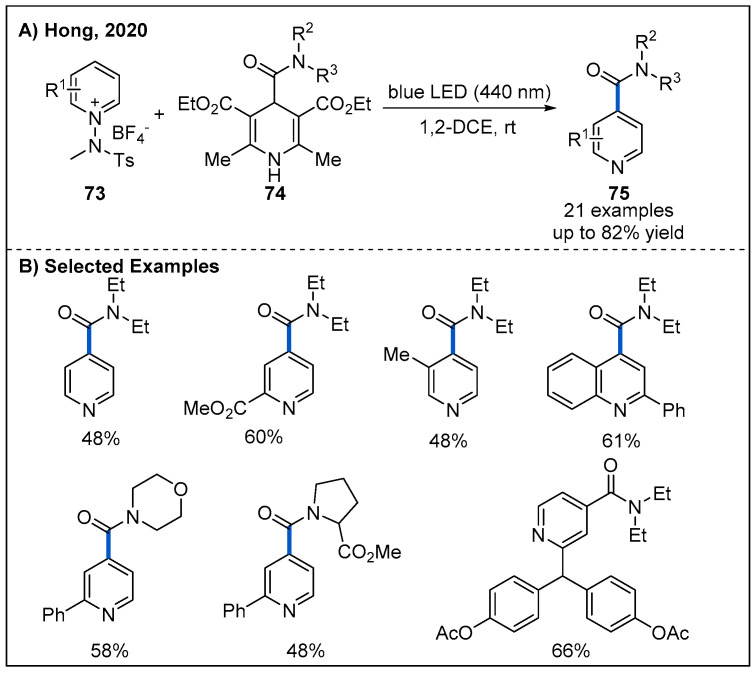
Photoinduced electron donor−acceptor (EDA)-enabled functionalization of pyridinium derivatives with 4-carbamoyl-1,4-dihydropyridines.

By exploiting electron donor−acceptor (EDA) complexes formed between *N*-amidopyridinium salts **73** and 1,4-dihydropyridines **74**, Hong reported a robust route to access various functionalized pyridines **75** under visible-light irradiation without requiring an additional photocatalyst (Figure 24) [59]. In this process, a wide variety of functionalized pyridines **75** with amide motifs at the C4-position were obtained when suitable 4-carbamoyl-1,4-dihydropyridine derivatives were used as carbamoyl radical precursors.

## 5. Radical Aminocarbonylation

With the development of radical chemistry, CO-based radical carbonylations have also emerged as a practical and versatile strategy for the synthesis of carbonyl compounds [60,61,62,63,64]. Despite the numerous advances in transition metal-catalyzed CO-based aminocarbonylations [65,66,67], over the past few years, the application of visible-light-driven activation and photoredox catalysis to radical carbonylation has opened a new way for the synthesis of amides and received considerable attention from synthetic community. In this section, we will discuss the recent representative achievements made in this area.

**Figure 25:**
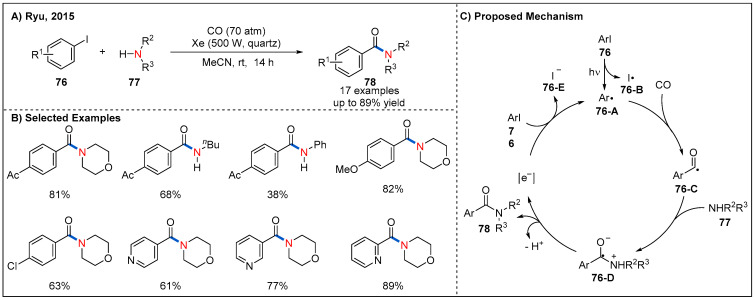
Photoinduced catalyst-free radical aminocarbonylation of aryl and heteroaryl iodides.

In 2015, Ryu’s group reported a photoinduced catalyst-free aminocarbonylation of aryl iodides with amines in the presence of CO gas by using Xe-lamp as a simple light source (Figure 25) [68]. This methodology shows broad substrate scope and good functional-group tolerance with respect to both components. Aromatic and heteroaromatic amides could be obtained in moderate to good yields. As for the mechanism, a chain process involving hybrid radical/ionic intermediates was proposed for the reaction (Figure 25C). Specifically, photoinduced cleavage of C-I bond of aryl iodide would yield an aryl radical **76-A** and iodine radical **76-B**. Then, **76-A** reacts with CO to generate acyl radical **76-C**, which could be captured by amines via a nucleophilic addition, providing a zwitterionic radical intermediate **76-D**. Finally, the electron transfer process between **76-D** and **76** would generate amide **78** and restart the radical chain reaction.

**Figure 26:**
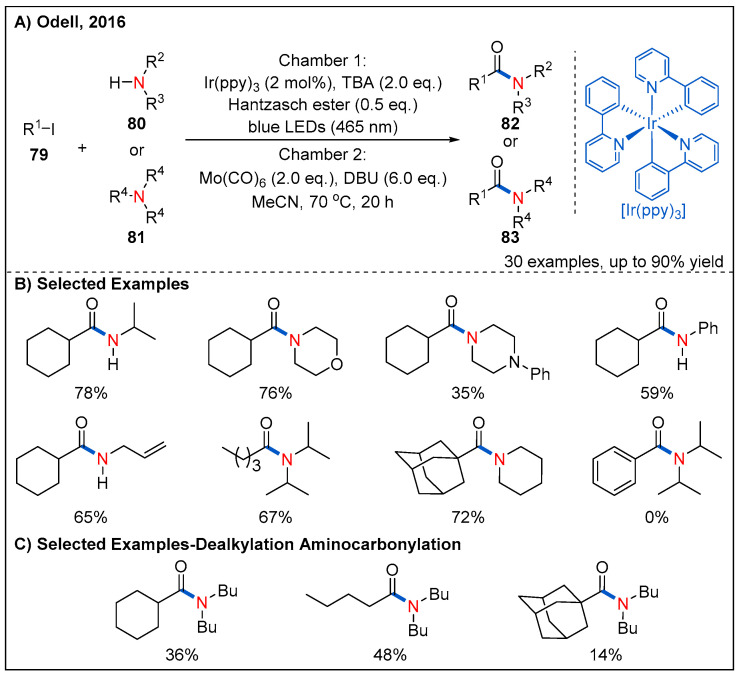
Photoinduced radical aminocarbonylation of unactivated alkyl iodides under low pressure of ex situ-formed CO.

In 2016, the Odell’s group utilized Mo(CO)_6_ as an efficient surrogate of CO and firstly developed an efficient, low-pressure visible-light-mediated radical aminocarbonylation of unactivated alkyl iodides **79** for the synthesis of alkyl amides (Figure 26) [69]. The reaction proceeded smoothly via a Ir(ppy)_3_-mediated SET process when using both tributylamine (TBA) and Hantzsch ester as co-reductants and alkyl iodides as oxidative radical precursors. Many amines, including primary and secondary alkyl amines, were well-tolerated. In addition, primary, secondary and tertiary alkyl iodides reacted with amines smoothly, furnishing the amide products in moderate to good yields. However, phenyl iodide was unsuccessful under the standard conditions. Remarkably, dealkylated amides **83** were successfully isolated in moderate yields when using tertiary amines with weaker nucleophilicity as reaction partners.

**Figure 27:**
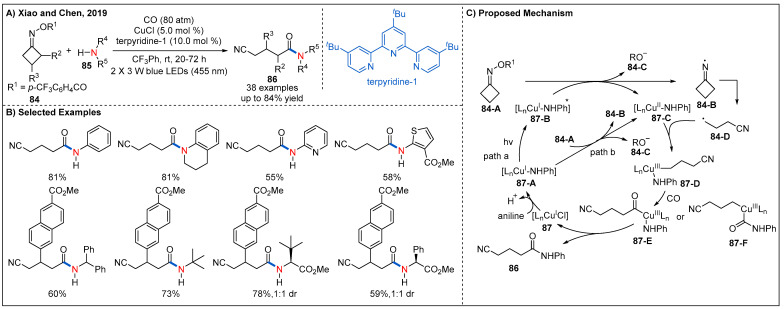
Visible-light-induced copper-catalyzed radical aminocarbonylation of redox-active cycloketone oxime esters.

The utility of earth-abundant metals such as copper for visible-light-assisted radical carbonylation reaction was another alternative way because the reserve of Ir and Ru were relatively low on Earth [70,71]. In 2019, Xiao and Chen described a copper-catalyzed radical aminocarbonylation of cycloketone oxime esters under visible-light photoredox catalysis to produce cyanoalkylated amides (Figure 27) [72]. A range of readily available aryl amines with electron-rich and electron-withdrawing substituents at the *ortho*-, *meta*-, and *para*-position, heteroaryl amines, and alkyl amines all participated in the reaction smoothly, providing the corresponding amides in moderate to good yields. In addition, a range of cyclobutanone oxime esters with various functional groups at the 2-position and 3-position are also well tolerated (Figure 27B). On the basis of a series of mechanistic experiments, two possible reaction pathways for the generation of cyanoalkyl radicals from cycloketone oxime esters are proposed (Figure 27C). Specifically, reduction of cyclobutanone oxime ester **84-A** by the photoexcited coppercomplex **87-B** (path a) or the ground state copper complex **87-A** (path b) via a SET process could give a cyclic iminyl radical **84-B** and the Cu(II) complex **87-C**. Then, the cyclic iminyl radical **84-B** undergoes a selective β−C-C bond scission to form the corresponding cyanoalkyl radical **84-D**, which can be intercepted by Cu(II) complex to form high-valent Cu(III) complex **87-D**. Subsequently, Cu(III) intermediate **87-D** undergoes further sequential coordination and insertion of CO to form the acyl copper intermediate **87-E** or **87-F**, which furnishes the final product **86-A** after reductive elimination and regenerates the active Cu(I) catalyst. Notably, in this process, the Cu(I) complex serves as both photocatalyst and cross-coupling catalyst. It not only accelerates the SET between oxime ester and copper complex under irradiation condition but also favors the chemoselective formation of amides rather than two-component coupling products-cyanoalkyl amines.

**Figure 28:**
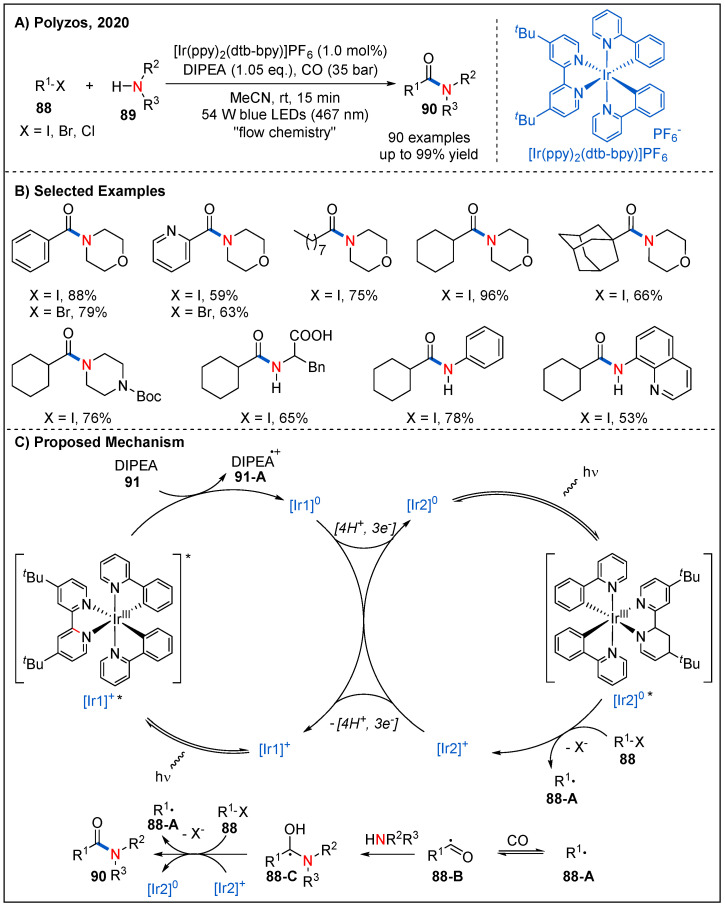
Photoinduced aminocarbonylation of aryl and alkyl halides by tandem photoredox catalysis.

In 2020, Polyzos and co-workers reported a novel strategy for aminocarbonylation of aryl and alkyl halides **88** with amines **89** by visible-light-driven tandem photoredox catalysis under continuous-flow conditions (Figure 28) [73]. In this strategy, [Ir(ppy)_2_(dtbbpy)]PF_6_ was used as a photocatalyst, and DIPEA as a sacrificial reductant. This catalytic system tolerates a wide range of halides. Aromatic and heteroaromatic bromides, iodides and chlorides all reacted smoothly with morpholine to furnish the expected amides **90** in good yields. Alkyl iodides also participated well in this reaction. On the other hand, cyclic or acyclic alkyl amines and aromatic amines were all well tolerated, giving the desired products in good yields. Remarkably, the reaction was also successfully applied to the construction of some pharmaceutically relevant amides. The use of continuous flow technique also affords operational ease, safety and scalability suitable for potential use in both academic and industrial laboratories. On the basis of detailed experimental studies, a plausible mechanism is proposed (Figure 28C). First, visible-light excitation of photocatalyst [Ir1]^+^ complex generates excited state [Ir1]^+*^ and undergoes reductive quenching with DIPEA to generate the reduced form [Ir1]^0^ and DIPEA radical cation **91-A**. Next, [Ir1]^0^ undergoes a hydrogen atom transfer (HAT) process with DIPEA radical cation **91-A** to form [Ir2]^0^. The [Ir2]^0^ species containing a semi-saturated (dtbbpy) ligand could initiates the second Ir photocatalytic cycle (Ir2). Namely, photoexcited [Ir2]^0*^ has a strong reduction potential (*E*^o^[Ir2] + /[Ir2]^0*^) = −3.0 to −1.70 V vs. SCE), which can be subsequently oxidized by organic halides with low oxidation potential to generate the corresponding aryl or alkyl radical **88-A** and [Ir2]^+^. The catalytic cycle is closed by the reduction of [Ir2]^+^ by [Ir1]^0^ or DIPEA. Finally, [Ir1]^+^ may be regenerated by a following hydrogen atom transfer from [Ir2]^+^ to acceptors such as aryl and alkyl radicals or the acyl radical.

The challenges in palladium-catalyzed carbonylation reaction of alkyl halides include the relative reluctance of sp^3^-hybridized electrophiles toward oxidative addition and the susceptibility of alkyl metal species to facile isomerization and β-hydrogen elimination [63]. To resolve these problems, Alexanian’s group applied electron-rich N-heterocyclic carbene ligand IMes (IMes = N,N’-bis(2,4,6-trimethylphenyl)imidazol-2-ylidene) at elevated temperature to achieve C-C bond construction from unactivated alkyl halides [74]. Key to the success of this strategy was the implementation of new modes of hybrid organometallic−radical reactivity in catalysis. Generation of carbon radicals from alkyl halides using such strategy offers a solution to challenges associated with the application of alkyl electrophiles in classical two-electron reaction modes. Moreover, Ryu’s group developed a practical and efficient room-temperature light/Pd-combined strategy to overcome this challenge [63]. Nevertheless, these reactions often require strong nucleophiles to promote the reductive elimination of palladium. Palladium-catalyzed carbonylation provides an effective way for the synthesis of acid chlorides, which can be converted into various carbonyl derivatives. However, sterically hindered ligands suitable for the reductive elimination often limit oxidative addition to aryl iodides and bromides and need high temperatures. Thus, the carbonylation of challenging electrophiles and nucleophiles remains a challenging synthetic task.

**Figure 29:**
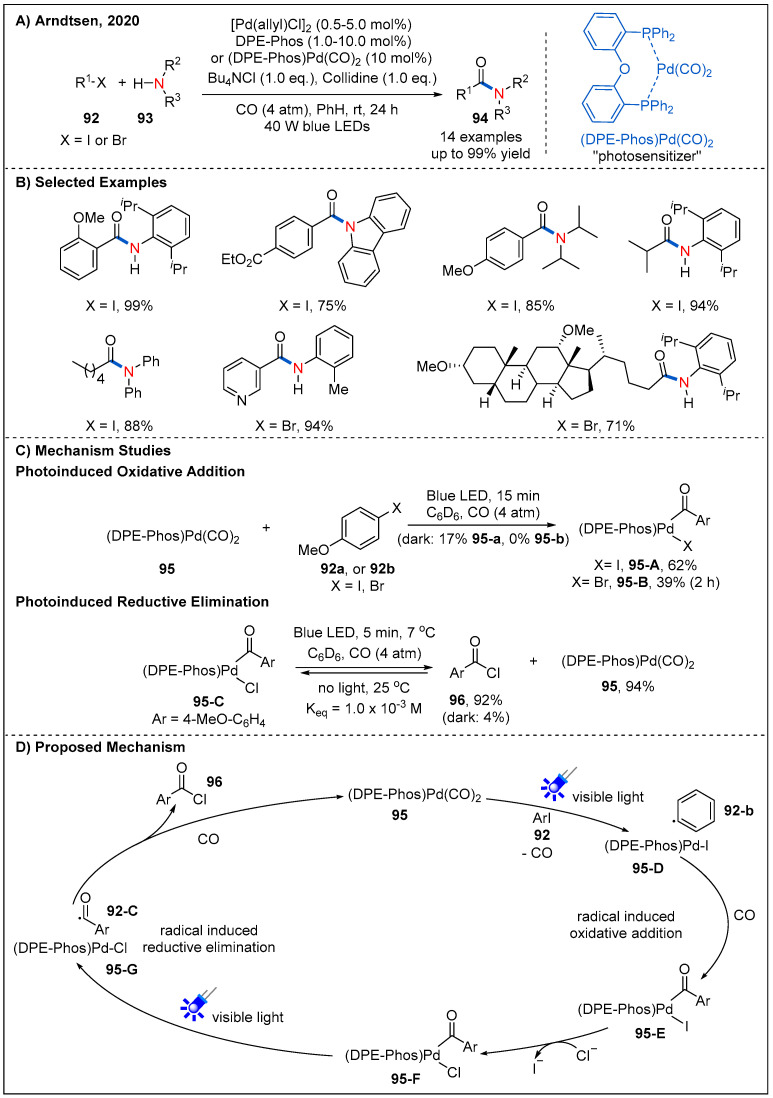
Photoinduced palladium-catalyzed aminocarbonylation of aryl and alkyl halides.

Recently, the Arndtsen’s group developed an elegant strategy that involves visible-light excitation of a palladium catalyst to drive both oxidative addition and reductive elimination steps with low barriers (Figure 29) [75,76]. Firstly, they discovered that a mixture of DPE-phos and [Pd(allyl)Cl]_2_ in the presence of blue-light irradiation could effectively promote the reaction of o-tolyl iodide and Bu_4_NCl to form acid chloride under the low pressure of CO (4 atm). Subsequently, they demonstrated that DPE-phos-ligated Pd(II)-acyl complex **95-C** could absorb blue light (λabs = 330 to 460 nm), leading to the near-quantitative reductive elimination to form acid chloride **96** within 5 min at low temperature. The reaction mixture reverts back to a near 1:1 equilibrium mixture of **95-C** and acid chloride **96** in the absence of light. It clearly confirms that light can promote the reductive elimination of acyl palladium species **95-C** at lower temperatures. In addition, experiments show that (DPEphos)Pd(CO)_2_ **95** can also absorb blue light (λabs = 300 to 420 nm) to result in the rapid oxidative addition of aryl iodides, aryl bromides, and even alkyl iodides in the presence of irradiation (Figure 29C). However, these processes are greatly inhibited or even unresponsive in the absence of visible-light irradiation, clearly demonstrating visible light plays an important role in the oxidative addition step. This strategy provides a gentle and effective way for the carbonylation of challenging electrophiles and nucleophiles.

The above catalytic system shows good functional-group tolerance and wide substrate scope. Aryl iodides could even react with sterically hindered amines to generate challenging amides in good yields. In addition, a range of alkyl iodides and primary alkyl bromides with low reduction potentials were also successfully converted into the corresponding desired products at low pressure of CO and temperatures. This light-driven palladium catalyst system also provides an effective method for the modification of some biologically important molecules. A plausible mechanism was proposed according to several mechanistic experiments (Figure 29D). Light-induced SET from (DPE-phos)Pd(CO)_2_ complex **95** to aryl or alkyl halides generates palladium specie **95-D** and the corresponding aryl radicals or alkyl radicals **92-B**. Both of them react with CO to form acyl palladium complex **95-E**; this complex then undergoes an ion exchange with chloride ion to release complex **95-F**. Finally, radical-induced reductive elimination of complex **95-G** occurs smoothly to deliver acyl chloride **96** under visible-light irradiation. This catalytic system represents a great breakthrough in the field of carbonylation chemistry since it opened a new way for the carbonylation of less-reactive electrophiles and weak nucleophiles at low temperatures and pressure of CO.

**Figure 30:**
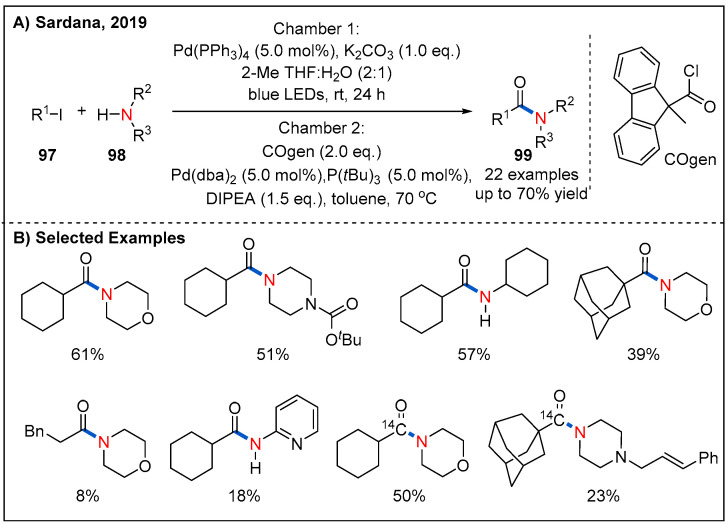
Amide synthesis via photoinduced palladium-catalyzed aminocarbonylation.

In 2019, the Sardana’s group disclosed a visible-light-mediated palladium-catalyzed aminocarbonylation of unactivated alkyl iodides **97** with stoichiometric amounts of carbon monoxide (Figure 30) [77]. In this approach, they used a two-chamber (chamber A and B) vial system. Specifically, the alkyl iodide, amine, Pd(PPh_3_)_4_, and potassium carbonate were added in chamber A under blue LED-light irradiation. On the other hand, chamber B was loaded with COgen and a palladium catalyst to release the CO. A range of primary, secondary, and tertiary alkyl iodides and morphine participated in the reaction smoothly to afford the corresponding products in moderate to good yields. Notably, the reaction provides a mild and efficient method [carbonyl-^13/14^C] alkyl amides while reducing radioactive waste.

**Figure 31:**
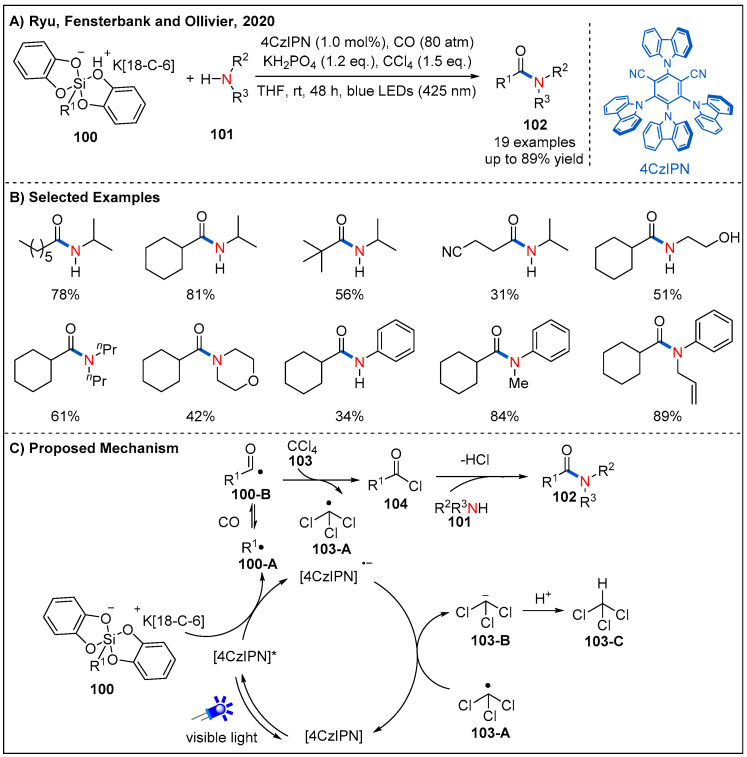
Visible-light-driven photoredox-catalyzed oxidative aminocarbonylation of alkyl silicates.

In 2020, Ryu, Fensterbank, Ollivier, and co-workers disclosed a visible-light-mediated CO-based oxidative aminocarbonylation reaction between alkyl (bis)catecholatosilicates and amines using carbon tetrachloride as both an external oxidant and chlorinating agent (Figure 31) [78]. By employing 4CzIPN as a photocatalyst, a range of aliphatic silicates were well tolerated. Primary, secondary and tertiary alkyl radicals derived from silicates undergo radical aminocarbonylation in the presence of carbon monoxide, carbon tetrachloride and various amines, furnishing the corresponding amides in generally good yields (Figure 31B). On their previous studies [79], the authors proposed a plausible mechanism for the reaction (Figure 31C). Specifically, photo-excited 4CzIPN* undergoes reductive quenching with alkyl silicates to generate 4CzIPN^•−^ and an alkyl radical **100-A**, which reacts with CO to form acyl radical **100-B**. Subsequently, acyl radical **100-B** abstracts a chlorine atom from carbon tetrachloride, furnishing acyl chloride **104** and trichloromethyl radical **103-A**. Nucleophilic attack of amine to acyl chloride delivers the desired amide product. At the same time, trichloromethyl radical **103-A** reacts with 4CzIPN^•−^ and then regenerates 4CzIPN and trichloromethyl anion **103-B**, finishing the photocatalytic cycle. Finally, the trichloromethyl anion abstracts a proton to release chloroform.

**Figure 32:**
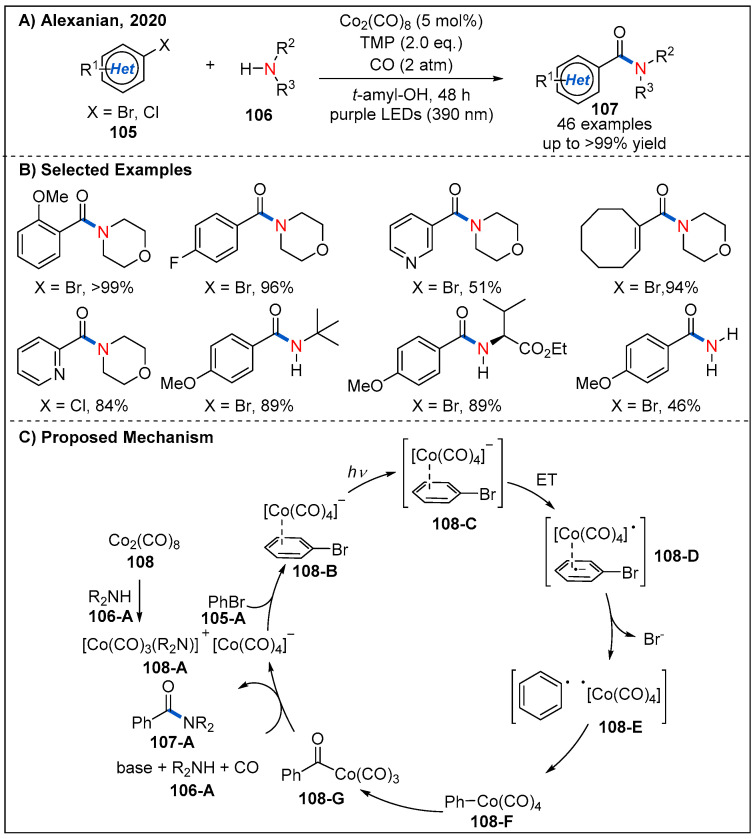
Visible-light-promoted cobalt-catalyzed aminocarbonylation of (hetero)aryl halides.

In 2020, Alexanian’s group reported a generally applicable visible-light-driven cobalt-catalyzed aminocarbonylation of (hetero)aryl halides (Figure 32) [80]. The use of simple carbonyl complex Co_2_(CO)_8_ in conjunction with carbon monoxide (2 atm) under visible light irradiation (390 nm LEDs) enabled the synthesis of the valuable amides in good to quantitative yields. Aryl and heteroaryl bromides bearing electron-donating and electron-withdrawing groups are all well-tolerated. Notably, aryl chlorides are also compatible with the reaction, providing the desired products in moderate to good yields.

A plausible catalytic cycle is depicted in Figure 32C. The active cobaltate **108-A** was first formed by disproportionation upon addition of amine or TMP to octacarbonyldicobalt. Next, **108-A** then coordinates to the (hetero)aryl halides **105-A** to form donor-acceptor complex **108-B**, which undergoes a reversible-light-promoted charge transfer. Loss of the bromide leads to a radical pair **108-E** that recombines in the solvent cage to produce a (hetero)aryl or vinyl cobalt species **108-F**. Finally, intermediate **108-F** undergoes CO migratory insertion to generate an acylcobalt species **108-G**, which is then substituted by the amine nucleophile to form the amide product with regeneration of the catalyst.

## 6. Beckmann Rearrangements

The Beckmann rearrangement is a classic reaction for the synthesis of amides and lactams starting from the readily available oximes [81]. However, the traditional Beckmann rearrangement typically requires strong acids and dehydrating agents at elevated temperature, producing an enormous amount of waste. The first photochemical Beckmann rearrangement was observed by De Mayo and co-workers in 1963, which utilized ultraviolet (UV) light source [82]. To make such rearrangement more economical and sustainable, recently, several visible-light-driven efficient and mild methods have been developed.

**Figure 33:**
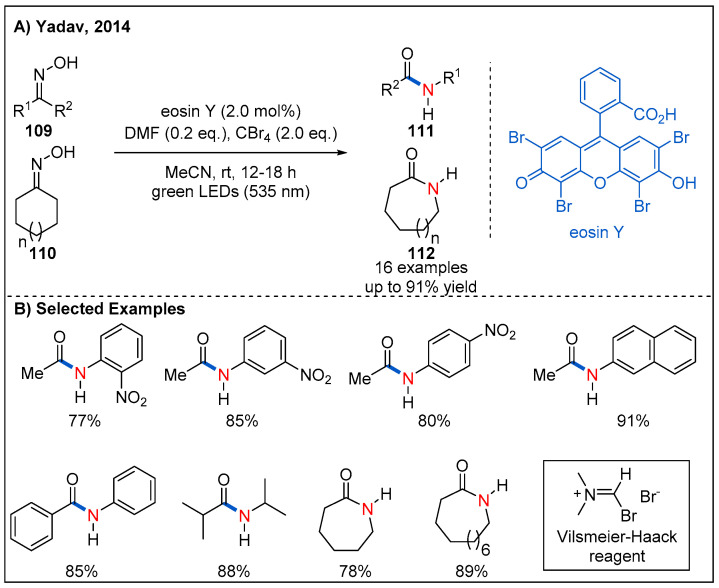
Photoinduced Beckmann rearrangement.

In 2014, Yadav’s group reported an efficient visible-light-induced Beckmann rearrangement of oximes using the Vilsmeier–Haack reagent, which is produced in situ by visible-light-driven photoredox-catalyzed reaction of carbon tetrabromide with *N,N*-dimethylformamide (DMF) (Figure 33) [83]. Inexpensive organic photocatalyst Eosin Y was identified to be the best photocatalyst [84], and the reactions of a range of aromatic, aliphatic acyclic and aliphatic cyclic oximes proceeded smoothly to provide the corresponding amides or lactams in generally good yields (Figure 33B). This indirect photocatalytic protocol is operationally simple and avoids the need for any corrosive, water-sensitive reagents and elevated temperatures.

**Figure 34:**
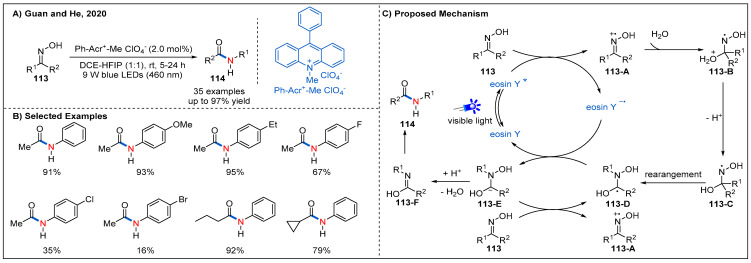
Visible-light-driven photoredox-catalyzed Beckmann rearrangement.

In 2020, Guan and He reported a more atom-economical and novel visible-light-induced Beckmann rearrangement of oximes using organic photocatalyst under mild conditions (Figure 34) [85]. The best reaction conditions involved using an inexpensive 10-methyl-9-phenylacridinium perchlorate as photocatalyst. The co-solvent HFIP was essential for this transformation due to its strong hydrogen-bonding ability. In this process, the presence of an electron-withdrawing group in the aryl moiety of the oximes appeared to decrease the yield, while the yield was enhanced in the case of the presence of electron-donating group-substituted substrates. Various alkyl aryl ketoximes and diaryl ketoximes can be effectively converted into the corresponding amides, while dialkyl oximes were not suitable for this reaction. On the basis of several control experiments, a plausible mechanism was proposed (Figure 34C). Firstly, oxidation of oxime **113** by visible-light-excited photoredox catalyst produces iminoxyl radical cation **113-A**. Then, nucleophilic attack of water on **113-A** and subsequent deprotonation generate nitrogen centred radical **113-C**. Next, **113-C** undergoes a 1,2-rearrangement to deliver intermediate **113-D**, which can be converted to intermediate **113-E** through a SET process with the reduced state of photocatalyst or **113** via a radical chain process. Finally, amides **114** were generated after losing a OH anion of **113-E** and subsequent tautomerization.

The obvious feature of the classic Beckman rearrangement is that the groups in the transposition of the hydroxyl group would preferentially migrate. On the other hand, general synthetic methods of oxime generate thermodynamically preferred *E* isomer or provide mixtures of *E* and *Z* isomers. Therefore, in this rearrangement reaction, the group with larger steric hindrance preferentially migrates; otherwise the reaction would often lead to a mixture of two amides. To realize the migration of groups with less steric hindrance, it is necessary to synthesize *Z* isomer, which was thermodynamically unstable. Thanks to the recent development of the photoisomerization of olefins [86], the photoisomerization of oximes has witnessed significant progress [87]. 

**Figure 35:**
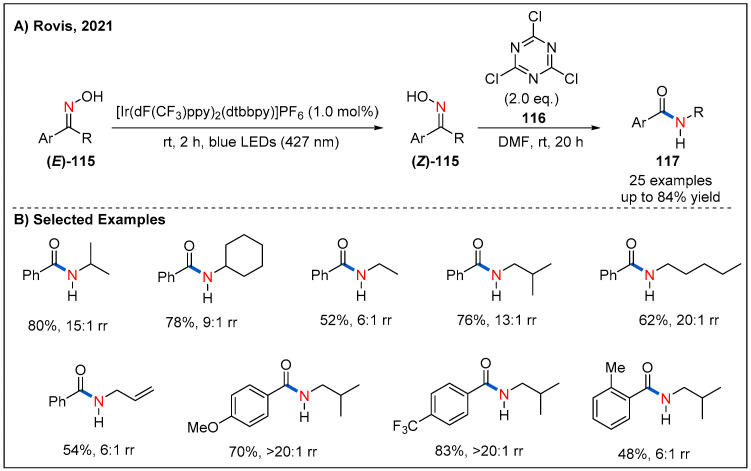
Visible-light-promoted nonclassical Beckmann rearrangement.

Very recently, the Rovis’ group realized the synthesis of *Z* isomer of oxime by photoisomerization via visible-light-mediated energy transfer catalysis (Figure 35) [88]. They found that [Ir(dF(CF_3_)ppy)_2_(dtbbpy)]PF_6_ was the best of choice because of its high triplet-state energy and long excited-state lifetime [89]. In addition, irradiation with 427 nm blue LEDs led to good *E/Z* ratios. Based on these results, they successfully achieved an efficient one-pot photoisomerization/Beckmann rearrangement of oximes **115**, namely, non-classical Beckmann rearrangement. Under the optimized conditions, a wide range of acyclic and cyclic oximes undergo the rearrangement with good regioselectivity to produce the corresponding amide products **117** in moderate to good yields. This one-pot isomerization/Beckmann rearrangement brings new regioselectivity to the Beckmann rearrangement, with the migration of alkyl groups instead of competing aryl groups.

## 7. Miscellaneous Amidation Methods

**Figure 36:**
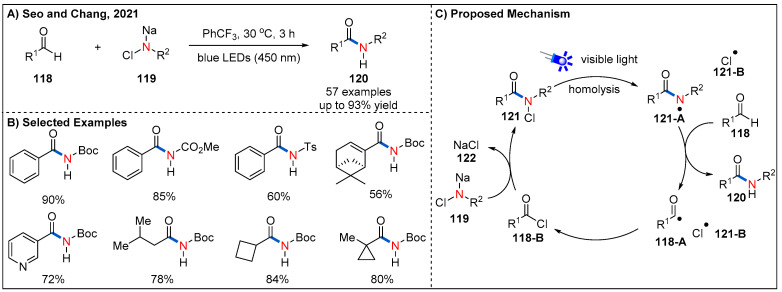
Visible-light-promoted *N*-chloro-*N*-sodio carbamate-mediated amidation of aldehydes.

Recently, Seo and Chang demonstrated that nitrogen-centered radicals [90,91,92], photocatalytically generated from the initially photostable *N*-chloro-*N*-sodio carbamates under irradiation of blue LEDs, could activate aldehydes to further enable amide synthesis (Figure 36) [93,94]. In this process, photocatalyst, external oxidant or coupling reagent are not required; *N*-chloro-*N*-sodio carbamates serve as a practical amidating source. A broad range of (hetero)aromatic and aliphatic aldehydes could be well accommodated, delivering diversely functionalized *N*-protected amides in generally good yields. On the basis of a series of control experiments, the authors have proposed a possible relay process-based mechanism for the reaction (Figure 36C). Key to the success of the reaction is the slow incorporation of photoactive *N*-chloroamide radical precursor **121** by the reaction between *N*-chloro-*N*-sodio-carbamate salt **119** and in situ-generated acid chloride intermediate **118-B**. Then, the intermediate **121** undergoes N−Cl bond homolysis to generate *N*-centered radical **121-A** under irradiation of blue LEDs. Hydrogen atom transfer (HAT) of the aldehydic C−H to the *N*-centered radical **121-A** leads to corresponding product **120**, while the translocation of *N*-radical to the *C*-radical. Finally, acyl radical **118-A** and chlorine radical recombine to generate acid chloride **118-B** to iterate the relay cycle. The authors further improved the sustainability of this amidation method by replacing α,α,α-trifluorotolune with ethyl acetate as an alternative green solvent.

**Figure 37:**
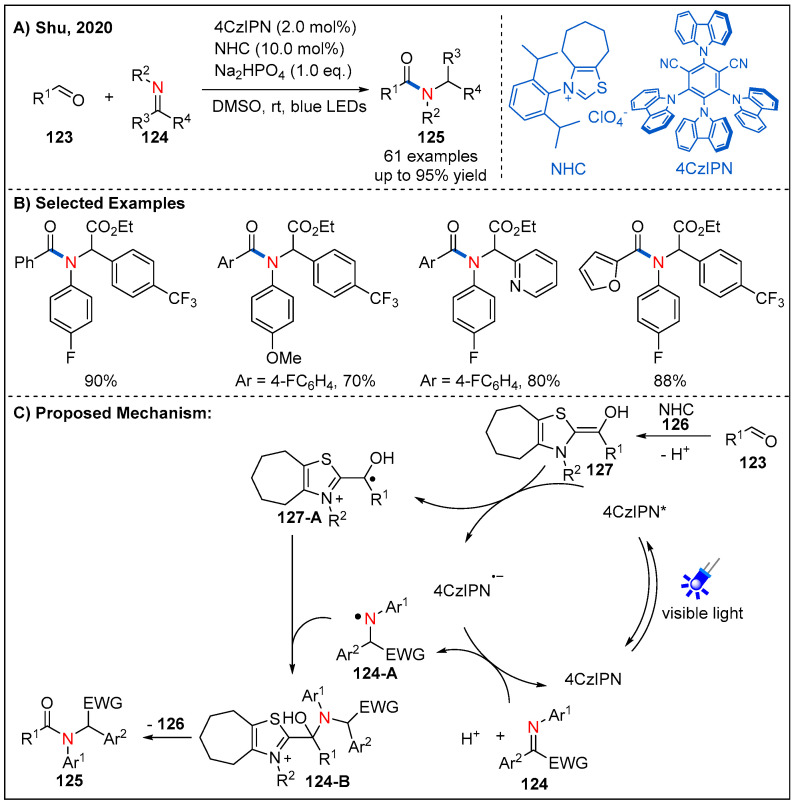
Visible-light-driven dual photoredox and N-heterocyclic carbene-catalyzed C-N bond-forming reaction for amide synthesis from aldehydes and imines.

In 2020, Shu’s group reported a 100% atom-economical and metal-free direct synthesis of amides from aldehydes **123** and imine esters **124** by the dual *N*-heterocyclic carbine and photoredox catalysis under redox-neutral conditions (Figure 37) [95]. In this dual catalytic strategy, photoredox catalysis enables the umpolung single-electron reduction of the imino ester to afford an *N*-centered radical, while the umpolung of aldehydes enabled by *N*-heterocyclic carbene generates a *C*-centered radical. Under the optimized conditions, a range of alkyl, aromatic and heteroaromatic aldehydes could be converted to the corresponding amides in consistently good yields.

On the basis of a range of mechanistic investigations, a plausible reaction mechanism is proposed as depicted in Figure 37C. Firstly, aldehyde condensed with the *N*-heterocyclic carbene catalyst to generate the corresponding Breslow intermediate **127**, which could undergo single-electron oxidation by the excited photocatalyst 4CzIPN* to give radical cationic intermediate **127-A** and reduced photocatalyst 4CzIPN^•−^. Next, the reduced photocatalyst 4CzIPN^•−^ could reduce imine **124** to give the *N*-centered radical **124-A** and 4CzIPN by SET and protonation. Intermediates **124-A** and **127-A** would undergo C-N bond-forming radical−radical cross-coupling to give **124-B**, which delivered the final amide product by regeneration of the *N*-heterocyclic carbene catalyst.

**Figure 38:**
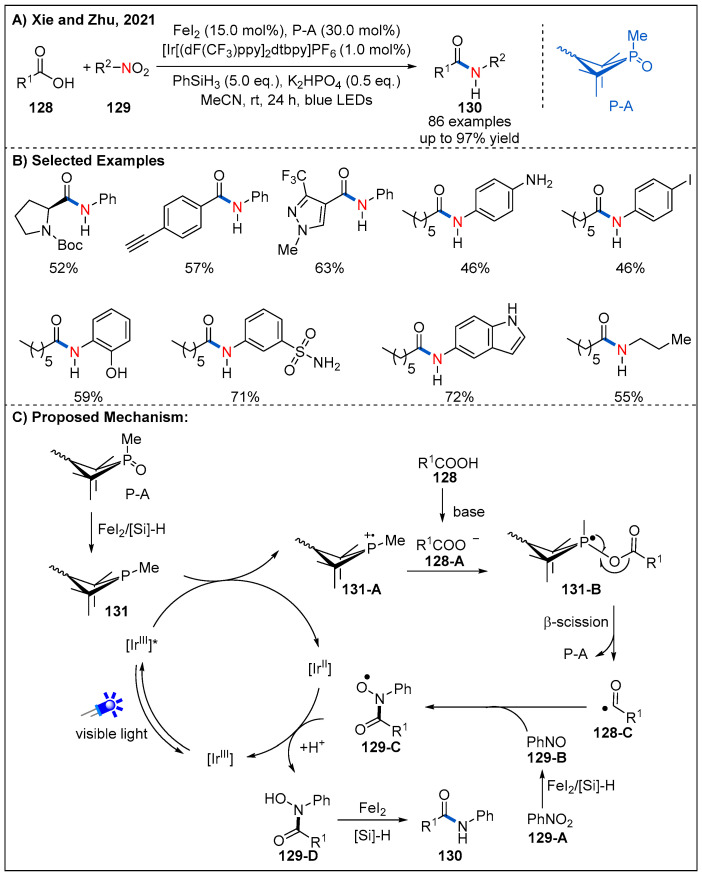
Umpolung amidation of carboxylic acids with nitroarenes and nitroalkanes.

As part of their continuing studies on development of Ph_3_P radical cation-mediated deoxygenation of carboxylic acids, Xie and Zhu recently described an impressive site-specific umpolung amidation of carboxylic acids **128** with nitroarenes and nitroalkanes **129** by combining FeI_2_, P(V)/P(III) and visible-light photoredox catalysis in a triplet synergistic manner (Figure 38) [96]. The reaction provides a novel approach to amide synthesis. Compared with conventional amidation methods, this protocol is characterized by replacement of amines with nitroarenes and thus tolerates several sensitive nucleophilic substituents. A number of nitroarenes bearing nucleophilic functional groups, such as free amino, hydroxy, and NH-free indole were all tolerated.

As depicted in Figure 38C, photoexcited [Ir(dF(CF_3_)ppy)_2_(dtbbpy)]^+*^ undergoes a SET-oxidation of **131**, which can be formed in situ by reduction from the precatalyst **P-A** in the presence of PhSiH_3_ and FeI_2_. This process leads to the formation of phosphine radical cation species **131-A** and [Ir(dF(CF_3_)ppy)_2_(dtbbpy)]^0^. Next, the phosphine radical cation **131-A** combines with carboxylate anion **128-A** to generate the radical intermediate **131-B**, which subsequently undergoes a β-C-O bond scission to form nucleophilic acyl radical **128-C** and complete the organophosphine catalytic cycle. At the same time, nitrosobenzene **129-B** was produced by the reduction of nitrobenzene **129-A** in the presence of FeI_2_ and PhSiH_3_. Nitrosobenzene **129-B** tends to trap acyl radical **128-C** to give rise to radical intermediate **129-C**. Single-electron oxidation of [Ir(dF(CF_3_)ppy)_2_(dtbbpy)]^0^ by radical intermediate **129-C** would generate intermediate **129-D** and complete the photoredox cycle. Finally, the reduction of intermediate **129-D** with FeI_2_/PhSiH_3_ releases the desired amide product **130**.

**Figure 39:**
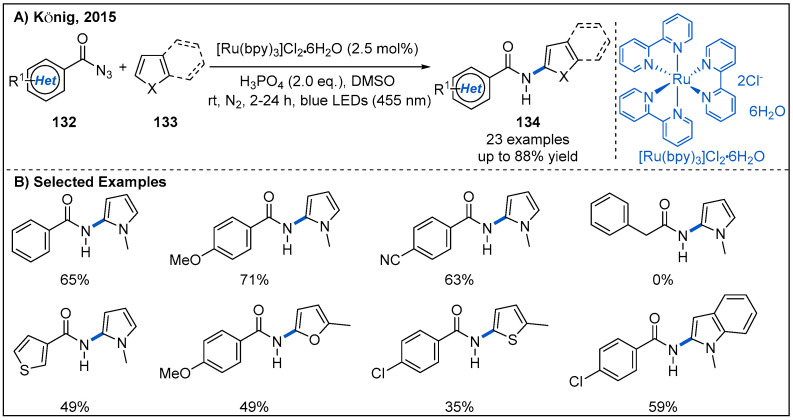
Visible-light-driven photoredox-catalyzed C-H amidation of heteroarenes with benzoyl azides.

Direct C-H amidations of heteroarenes or arenes is an alternative method to amide synthesis, but a drawback of this intriguing strategy is the typically required harsh reaction conditions. In 2015, the König group disclosed a visible-light-mediated C-H amidation of heteroarenes with benzoyl azides using [Ru(bpy)_3_]Cl_2_ as photocatalyst (Figure 39) [97]. Interestingly, the authors noted that the addition of stoichiometric phosphoric acid as an additive is essential for the formation of the desired amidation product. The benzoyl nitrene, produced from the decomposition of azides, may be protonated in the presence of strongly acidic phosphoric acid to give electrophilic nitrenium ions. Then, electrophilic nitrenium ions react with the electron-rich heteroarene. Benzoyl azides bearing electron-donating groups or electron-withdrawing groups at the aromatic ring were tolerated (Figure 39B). However, alkyl, phenyl, diphenylphosphoryl or benzyl acyl azides **132** proved to be not suitable for this reaction. The control experiments revealed that this C-H amidation process is completely shut down, when either the visible light or the photocatalyst is excluded, confirming the photoredox characteristics of this transformation.

**Figure 40:**
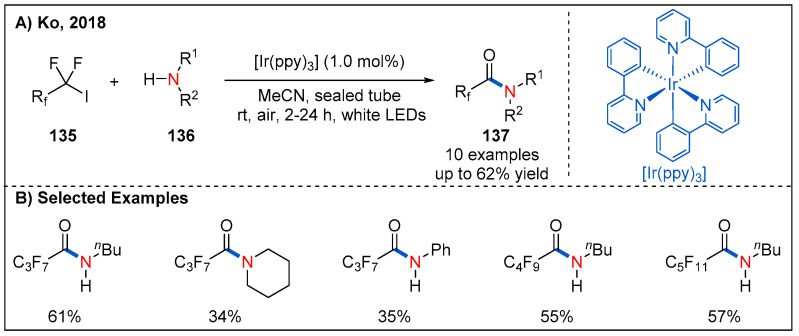
Photoredox-catalyzed synthesis of fluorinated alkyl amides from perfluorinated alkyl iodides and amines.

Fluorinated compounds have been widely used in pharmaceutical chemistry. Given the significance of this structural motif, in 2018, Ko and co-workers reported a visible-light-induced method for the preparation of perfluorinated alkyl amides by reaction between fluorinated alkyl halides and amines using *fac*-Ir(ppy)_3_ as a photocatalyst under aerobic conditions (Figure 40) [98].

**Figure 41:**
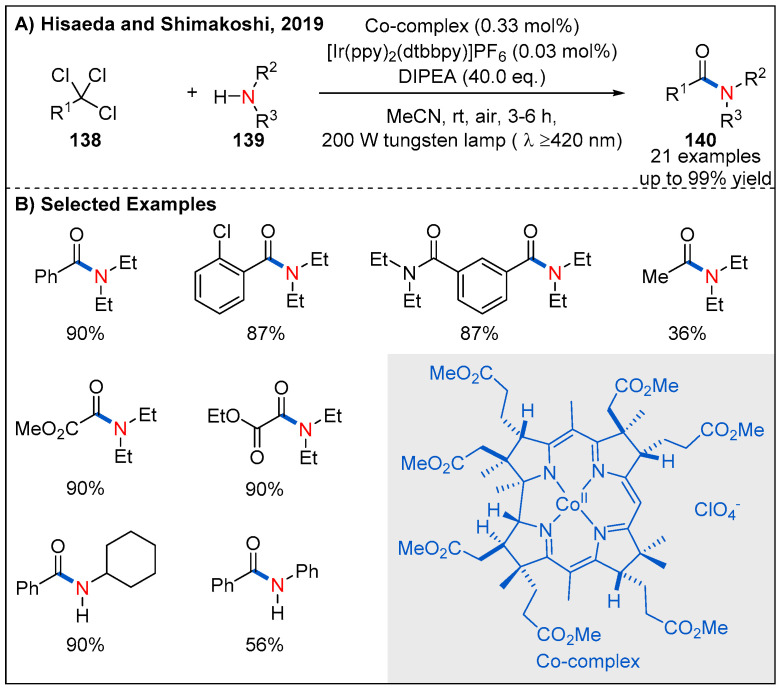
Visible-light-driven amide synthesis from trichlorinated compounds.

At almost the same time, Hisaeda and Shimakoshi’s described a visible-light-driven amide synthesis from trichlorinated organic compounds with the B_12_ complex as a catalyst and [Ir(dtbbpy)(ppy)_2_]PF_6_ as a photosensitizer (Figure 41) [99]. The reaction was performed under aerobic conditions and triethylamine was using as a sacrificial reagent. Under the optimal conditions, a range of trichlorinated compounds are proved to be well compatible with the reaction to form the corresponding amide products in generally excellent yields. Interestingly, this transformation allows access to useful α-ketoamides in high yields, when using suitable substrates such as ethyl trichloacetate.

**Figure 42:**
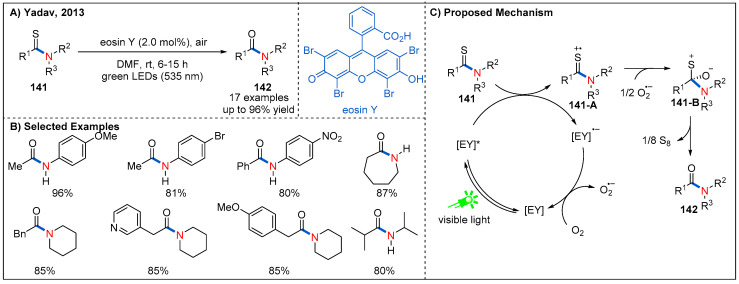
Visible-light-driven aerobic desulfurization–oxygenation of thioamides.

In 2013, Yadav and co-workers reported an aerobic desulfurization-oxygenation of thioamides **141** into amides **142** by employing eosin Y as an organic photoredox catalyst (Figure 42) [100]. In the cases of substrates containing alkyl and aryl substituents at the skeleton of thioamides, the reaction proceeded smoothly to give amides in good yields. The authors showed that elemental sulfur could be detected in this catalytic system. This result is consistent with the proposed mechanism (Figure 42C). First, visible-light-excited photocatalyst oxidized thioamides **141** to form **141-A**, and reduced-stated photocatalyst via a SET event. Then, the molecular oxygen oxidized the reduced-state photocatalyst to regenerate photocatalyst and close the cycle of photoredox catalysis. Nucleophilic addition of superoxide radical anion to thione cation radical **141-A**, generating zwitterion **141-B**. Finally, the elimination of S_8_ produces the amide product **142**.

**Figure 43:**
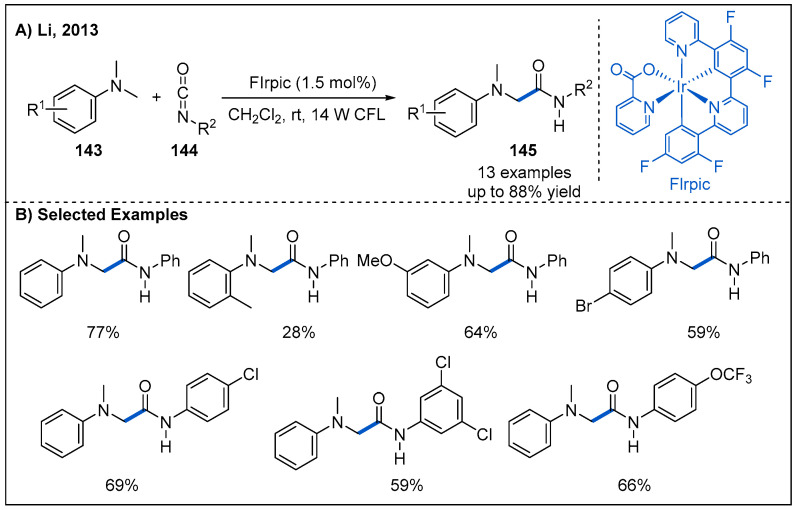
Visible-light-driven photoredox-catalyzed amide synthesis from isocyanates and *N,N*-dimethylaniline derivatives.

In 2013, Li and co-workers reported a visible-light-mediated radical addition reaction between *N,N*-dimethylaniline derivatives **143** and isocyanates **144** (Figure 43) [101]. After screening of six different photocatalysts, they identified bis[2-(4,6-difluorophenyl)pyridinato-C^2^,N](picolinato)iridium(III) (FIrpic) to be the best candidate. Mechanistically, the reaction involves photoredox-catalyzed generation of α-aminoalkyl radicals from *N,N*-dimethylaniline derivatives and subsequent addition to the electron-deficient carbon atom of the isocaynates as the key steps. Generally, the aromatic isocyanates bearing electron-donating and electron-withdrawing groups undergo the reaction efficiently to furnish the amide products in moderate to good yields. However, alkyl isocyanate did not give the desired α-amino amide under the current reaction conditions.

**Figure 44:**
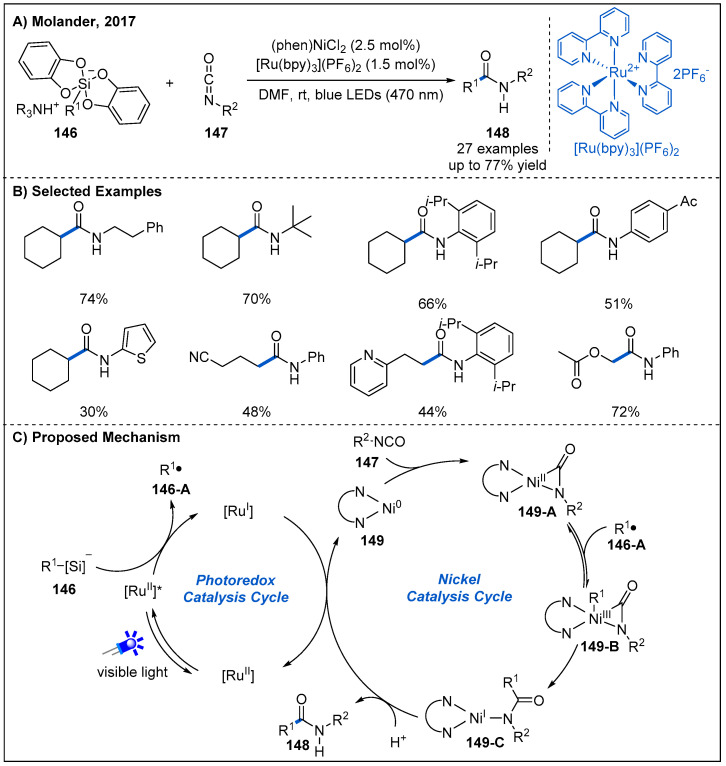
Amide synthesis by visible-light-driven dual nickel/photoredox-catalyzed coupling between alkylsilicates and alkyl/aryl isocyanates.

In 2017, Molander and co-workers detailed an interesting amidation reaction between alkylsilicate reagents **146** and alkyl/aryl isocyanates **147** by combining nickel and photoredox catalysis (Figure 44) [102]. This dual catalysis system shows good functional group tolerance with respect to both alkylsilicates and alkyl and aryl isocyanates, giving the corresponding alkyl amides in moderate to high yields. This protocol features mild reaction conditions and the absence of stoichiometric reductant. A possible mechanism involving a photoredox/nickel dual catalytic mode is proposed for the reaction (Figure 44C). Initially, Ni(II) carbonyl-amido intermediate **149-A** is formed upon oxidative addition of Ni(0) to the isocyanate, supported by the signal changes in the related NMR and IR spectra. Subsequently, Ni(III) complex **149-B** can be generated upon radical addition. Then, **149-B** undergoes reductive elimination to deliver Ni(I) complex **149-C**, followed by protonation to yield amide product **148**. The resulting Ni(I) complex is reduced by [Ru(bpy)]^1+^ to turn over both the nickel and photoredox catalytic cycles.

**Figure 45:**
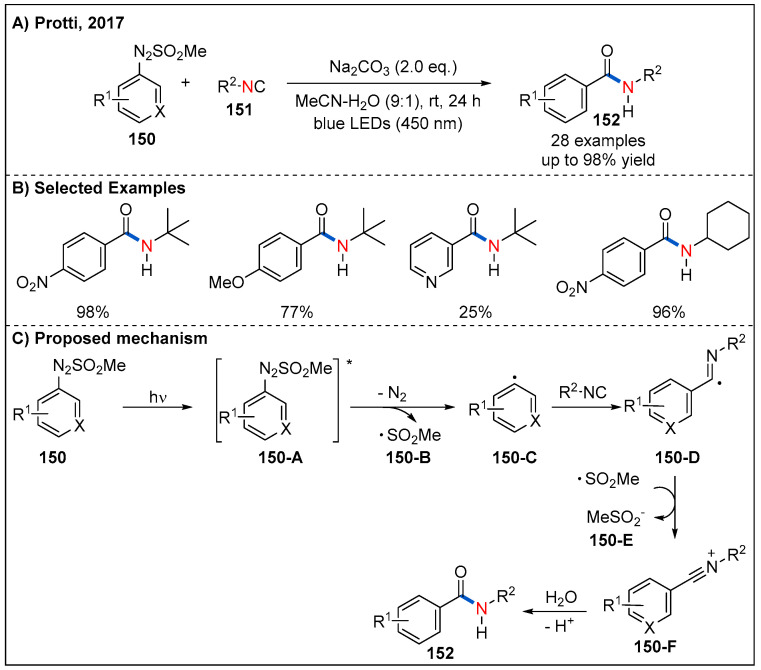
Visible-light-driven metal-free carbamoylation of arylazo sulfones with isocyanides.

Given the availability of arylazo sulfones from stable anilines, in 2017, Protti’s group reported a practical visible-light-driven, metal-free carboamidation of arylazo sulfone **150** derived aryl radical in the presence of isocyanides **151** in aqueous acetonitrile (Figure 45) [103]. Remarkably, compared to arenediazonium salts, arylazo sulfones show higher compatibility. However, the scope of isocyanide is quite limited. In this process, aryl radicals **150-C** can be generated smoothly by direct irradiation of arylazo sulfones **150** upon 410 nm LED irradiation, which undergoes a radical addition with isocyanides to form intermediate **150-D**. Subsequently, **150-D** is oxidized by **150-B**, giving an intermediate **150-F**. Finally, hydrolysis of **150-F** produces the corresponding aromatic amide products (Figure 45C).

**Figure 46:**
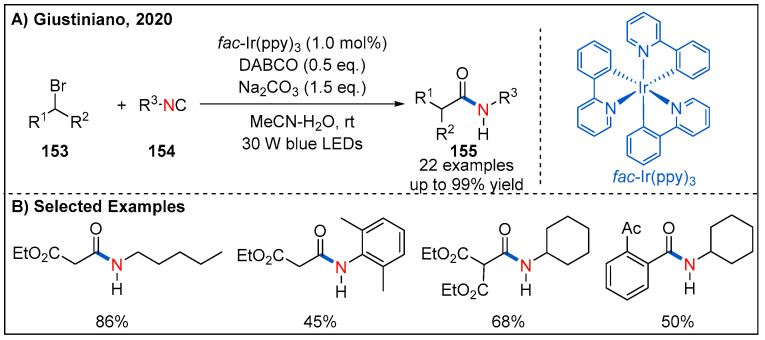
Visible-light-promoted photoredox-catalyzed amide synthesis from electron-poor alkyl/aryl bromides and isocyanides.

In 2020, Giustiniano and co-workers developed a visible-light-driven photoredox-catalyzed method for the formation of secondary amides from electron-poor organic bromides and isocyanides (Figure 46) [104]. In this process, treatment of organic bromides with isocyanides in the presence of *fac*-Ir(ppy)_3_, Na_2_CO_3_ and DABCO under irradiation of blue LED gave rise to amide products in good to high yields. The addition of a sacrificial electron donor, diazabicyclo[2.2.2]octane (DABCO), is key to improving the yield. In addition to working as a common base, it was reasoned that DABCO can also act as a sacrificial electron donor to facilitate regeneration of the Ir(III) photocatalyst. Interestingly, aryl bromides with an electron-withdrawing group at the phenyl ring are also compatible with reaction though with decreased yields of amide products.

**Figure 47:**
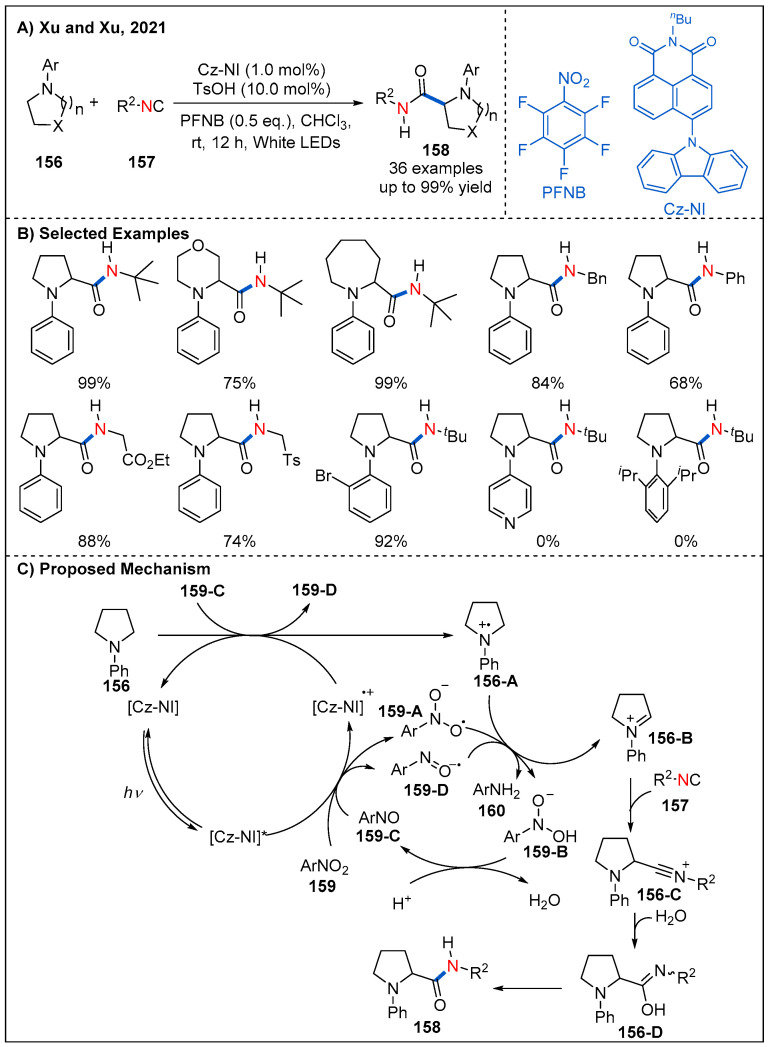
Visible-light-induced photoredox-catalyzed oxidative carbamoylation of saturated *aza*-heterocycles with isocyanides.

Recently, Xu and Xu’s group described a robust method for direct α-C(sp^3^)−H carbamoylation of saturated *aza*-heterocycles **156** with isocyanides **157** by using an elegantly designed organic photocatalyst under mild conditions (Figure 47) [105]. In the process of condition optimization, the author found that the structure of the photosensitizer has significant influence on the reaction. Finally, photocatalyst Cz-NI was identified to be the best one, which has a larger dihedral angle (65.3°), larger transition dipole moment and effective oxidative and reductive potential of the excited state; both features are critical to promoting this reaction. On the other hand, the choice of oxidant and co-catalyst is also very important. The optimal reaction conditions are using PFNB as oxidant and TsOH as co-catalyst. Nitrogen heterocycles of different sizes, including five-, six- and seven-membered rings, proved to be suitable and the corresponding products could be achieved in good to excellent yields. Larger rings such as eight, nine-membered rings are also tolerated and amides are obtained in moderate yields. The substituents on the nitrogen heterocycle have obvious influence on the reaction. When the phenyl group is replaced with pyridine or 2,6-diisopropylphenyl, the reaction is completely inhibited. A possible mechanism of this transformation is also proposed as shown in Figure 47C. Photoexcited [Cz-NI]* is oxidized by PFNB to form the [Cz-NI]^•+^ radical and anion **159-A**. Next, [Cz-NI]^•+^ is capable of accepting an electron from amine **156** to close the photoredox catalysis cycle and generates a nitrogen radical cation **156-A**. A hydrogen atom transfer (HAT) event between nitrogen radical cation **156-A** and radical anion **159-A** occurs to afford iminium ion **156-B** and intermediate **159-B**. Protonation of **159-B** in an acidic environment leads to its further conversion to pentafluoro-nitrosobenzene **159-C**, which is capable of accepting an electron from substrate amine **156** or undergoing a SET process with the reduced form of the photocatalyst. Finally, the desired amide product is formed through a nucleophilic attack of isonitrile onto iminium intermediate **156-C** and following Ugi-type reaction process.

## 8. Conclusions and Outlook

With the impressive involvement in the fields of photochemistry and radical chemistry, visible-light-driven photoredox catalysis has become one of the most powerful tools in organic synthesis. Over the past few years, visible-light-driven photoredox catalysis has been extensively applied to the synthesis of various structurally diverse amides. In this review, we summarized the recent representative examples in field of visible-light-mediated amide construction, which were discussed according to different catalytic modes and radical precursors. In contrast to traditional methods involving couplings of carboxylic acids with amines, photoredox catalysis allows the formation of amides under milder and more sustainable reaction conditions mainly via radical processes. The majority of these reactions often proceed at room temperature with good functional group tolerances. In some cases, stoichiometric coupling reagents are not required. Given the unique activation modes, this catalytic strategy further expands the range of substrates beyond the traditionally used carboxylic acids and amines. Aryl halides, alkyl halides, arenes and even alkanes, to name a few, can be used for the synthesis of diversely functionalized amides [106]. Actually, these advantages are mainly due to the characteristics of photoredox catalysis, wherein various radicals can often be generated in a controllable manner under low-energy visible-light irradiation. Moreover, photoredox catalysis provides an opportunity for the development dual catalysis systems, such as synergistic nickel and photoredox catalysis, and N-heterocyclic carbene and photoredox catalysis. These catalytic systems allow the formation of amides that are otherwise difficult to synthesize. Despite these advances, there are still many opportunities for the development of new and robust methods in this area. From the perspective of medicinal chemistry, the enantioselective synthesis of amides and complex peptide through photoredox catalysis is still highly desirable. On the other hand, most visible-light-mediated amidation reactions are performed at small-scale. Therefore, it is highly desirable to apply continuous-flow photochemistry in the area of amide synthesis [107]. We hope that this review will inspire further developments that would address the abovementioned challenges [108].

## Data Availability

All data are available in the manuscript.

## References

[1] De Figueiredo R.M., Suppo J.S., Campagne J.M. (2016). Nonclassical Routes for Amide Bond Formation. Chem. Rev..

[2] Bednarek C., Wehl I., Jung N., Schepers U., Brase S. (2020). The Staudinger Ligation. Chem. Rev..

[3] Massolo E., Pirola M., Benaglia M. (2020). Amide Bond Formation Strategies: Latest Advances on a Dateless Transformation. Eur. J. Org. Chem..

[4] Santos A.S., Silva A.M.S., Marques M.M.B. (2020). Sustainable Amidation Reactions-Recent Advances. Eur. J. Org. Chem..

[5] Lundberg H., Tinnis F., Selander N., Adolfsson H. (2014). Catalytic amide formation from non-activated carboxylic acids and amines. Chem. Soc. Rev..

[6] Carey J.S., Laffan D., Thomson C., Williams M.T. (2006). Analysis of the Reactions Used for the Preparation of Drug Candidate Molecules. Org. Biomol. Chem..

[7] Ghose A.K., Viswanadhan V.N., Wendoloski J.J. (1999). A Knowledge Based Approach in Designing Combinatorial or Medicinal Chemistry Libraries for Drug Discovery. 1. A Qualitative and Quantitative Characterization of Known Drug Databases. J. Comb. Chem..

[8] Todorovic M., Perrin D.M. (2020). Recent developments in catalytic amide bond formation. Pept. Sci..

[9] Sabatini M.T., Boulton L.T., Sneddon H.F., Sheppard T.D. (2019). A green chemistry perspective on catalytic amide bond formation. Nat. Catal..

[10] Valeur E., Bradley M. (2009). Amide bond formation: Beyond the myth of coupling reagents. Chem. Soc. Rev..

[11] Dunetz J.R., Magano J., Weisenburger G.A. (2016). Large-Scale Applications of Amide Coupling Reagents for the Synthesis of Pharmaceuticals. Org. Process Res. Dev..

[12] Sabatini M.T., Boulton L.T., Sheppard T.D. (2017). Borate esters: Simple catalysts for the sustainable synthesis of complex amides. Sci. Adv..

[13] Mylavarapu R.K., Gcm K., Kolla N., Veeramalla R., Koilkonda P., Bhattacharya A., Bandichhor R. (2007). Boric Acid Catalyzed Amidation in the Synthesis of Active Pharmaceutical Ingredients. Org. Process Res. Dev..

[14] Ciszewski L.W., Rybicka-Jasinska K., Gryko D. (2019). Recent developments in photochemical reactions of diazo compounds. Org. Biomol. Chem..

[15] Ford A., Miel H., Ring A., Slattery C.N., Maguire A.R., McKervey M.A. (2015). Modern Organic Synthesis with alpha-Diazocarbonyl Compounds. Chem. Rev..

[16] Hua T.-B., Yang Q.-Q., Zou Y.-Q. (2019). Recent Advances in Enantioselective Photochemical Reactions of Stabilized Diazo Compounds. Molecules.

[17] Kirmse W. (2002). 100 Years of the Wolff Rearrangement. Eur. J. Org. Chem..

[18] Prier C.K., Rankic D.A., MacMillan D.W.C. (2013). Visible Light Photoredox Catalysis with Transition Metal Complexes: Applications in Organic Synthesis. Chem. Rev..

[19] Skubi K.L., Blum T.R., Yoon T.P. (2016). Dual Catalysis Strategies in Photochemical Synthesis. Chem. Rev..

[20] Romero N.A., Nicewicz D.A. (2016). Organic Photoredox Catalysis. Chem. Rev..

[21] Xuan J., Zhang Z.-G., Xiao W.-J. (2015). Visible-Light-Induced Decarboxylative Functionalization of Carboxylic Acids and Their Derivatives. Angew. Chem. Int. Ed..

[22] Chen Y., Lu L.-Q., Yu D.-G., Zhu C.-J., Xiao W.-J. (2019). Visible Light-driven Organic PhotoChemical Synthesis in China. Sci. China Chem..

[23] Ren L., Ran M., He J., Qian Y., Yao Q. (2019). Recent Advance in the Transition-Metal Free Coupling Reactions for the Construction of C–X Bonds Induced by Light. Chin. J. Org. Chem..

[24] Wang P.-Z., Zhao Q.-Q., Xiao W.-J., Chen J.-R. (2020). Recent advances in visible-light photoredox-catalyzed nitrogen radical cyclization. Green Synth. Catal..

[25] Rao M., Wu W.-H., Yang C. (2021). Recent progress on the enantioselective excited-state photoreactions by pre-arrangement of photosubstrate(s). Green Synth. Catal..

[26] Zhao S.N., Wang G., Poelman D., Van Der Voort P. (2018). Metal Organic Frameworks Based Materials for Heterogeneous Photocatalysis. Molecules.

[27] Pawlowski R., Stanek F., Stodulski M. (2019). Recent Advances on Metal-Free, Visible-Light- Induced Catalysis for Assembling Nitrogen- and Oxygen-Based Heterocyclic Scaffolds. Molecules.

[28] Zhou L. (2021). Recent Advances in C-F Bond Cleavage Enabled by Visible Light Photoredox Catalysis. Molecules.

[29] Luu T.G., Jung Y., Kim H.K. (2021). Visible-Light-Induced Catalytic Selective Halogenation with Photocatalyst. Molecules.

[30] Liu J., Liu Q., Yi H., Qin C., Bai R., Qi X., Lan Y., Lei A. (2014). Visible-light-mediated decarboxylation/oxidative amidation of alpha-keto acids with amines under mild reaction conditions using O_2_. Angew. Chem. Int. Ed..

[31] Gaspa S., Farina A., Tilocca M., Porcheddu A., Pisano L., Carraro M., Azzena U., De Luca L. (2020). Visible-Light Photoredox-Catalyzed Amidation of Benzylic Alcohols. J. Org. Chem..

[32] Singha K., Ghosh S.C., Panda A.B. (2021). Visible Light-Driven Efficient Synthesis of Amides from Alcohols using Cu−N−TiO_2_ Heterogeneous Photocatalyst. Eur. J. Org. Chem..

[33] Nandi J., Vaughan M.Z., Sandoval A.L., Paolillo J.M., Leadbeater N.E. (2020). Oxidative Amidation of Amines in Tandem with Transamidation: A Route to Amides Using Visible-Light Energy. J. Org. Chem..

[34] Leow D. (2014). Phenazinium Salt-catalyzed Aerobic Oxidative Amidation of Aromatic Aldehydes. Org. Lett..

[35] Leung F.K.-C., Cui J.-F., Hui T.-W., Kung K.K.-Y., Wong M.-K. (2015). Photooxidative Amidation of Aldehydes with Amines Catalyzed by Rose Bengal. Asian J. Org. Chem..

[36] Wang X.-F., Yu S.-S., Wang C., Xue D., Xiao J. (2016). BODIPY Catalyzed Amide Synthesis Promoted by BHT and Air under Visible Light. Org. Biomol. Chem..

[37] Deng J.-R., Chan W.-C., Chun-Him Lai N., Yang B., Tsang C.S., Chi-Bun Ko B., Lai-Fung Chan S., Wong M.K. (2017). Photosensitizer-free Visible Light-mediated Gold-catalysed cis-Difunctionalization of Silyl-substituted Alkynes. Chem. Sci..

[38] Liu H., Zhao L., Yuan Y., Xu Z., Chen K., Qiu S., Tan H. (2016). Potassium Thioacids Mediated Selective Amide and Peptide Constructions Enabled by Visible Light Photoredox Catalysis. ACS Catal..

[39] Song W., Dong K., Li M. (2020). Visible Light-Induced Amide Bond Formation. Org. Lett..

[40] Cohen I., Mishra A.K., Parvari G., Edrei R., Dantus M., Eichen Y., Szpilman A.M. (2017). Sunlight Assisted Direct Amide Formation via A Charge-transfer Complex. Chem. Commun..

[41] Ragupathi A., Sagadevan A., Lin C.C., Hwu J.R., Hwang K.C. (2016). Copper(I)-catalysed Oxidative C-N Coupling of 2-Aminopyridine with Terminal Alkynes Featuring a C≡C Bond Cleavage Promoted by Visible Light. Chem. Commun..

[42] Pampana V.K.K., Sagadevan A., Ragupathi A., Hwang K.C. (2020). Visible Light-promoted Copper Catalyzed Regioselective Acetamidation of Terminal Alkynes by Arylamines. Green Chem..

[43] Wang D., Li J., Cai S., Chen J., Zhao Y. (2014). Visible Light Induced Photocatalytic Conversion of Enamines into Amides. Synlett.

[44] Guerinot A., Reymond S., Cossy J., Jiang D., He T., Ma L., Wang Z., Bolsakova J., Jirgensons A. (2012). Ritter Reaction: Recent Catalytic Developments. Eur. J. Org. Chem..

[45] Hari D.P., Hering T., Konig B. (2014). The Photoredox-catalyzed Meerwein Addition Reaction: Intermolecular Amino-arylation of Alkenes. Angew. Chem. Int. Ed..

[46] Li G.X., Morales-Rivera C.A., Gao F., Wang Y., He G., Liu P., Chen G. (2017). A Unified Photoredox-catalysis Strategy for C(sp^3^)-H Hydroxylation and Amidation Using Hypervalent Iodine. Chem. Sci..

[47] Zhang Y., Teuscher K.B., Ji H. (2016). Direct α-heteroarylation of Amides (α to nitrogen) And Ethers through A Benzaldehyde-mediated Photoredox Reaction. Chem. Sci..

[48] Cheng W.-M., Shang R., Yu H.Z., Fu Y. (2015). Room-Temperature Decarboxylative Couplings of α-Oxocarboxylates with Aryl Halides by Merging Photoredox with Palladium Catalysis. Chem. Eur. J..

[49] Shang R., Fu Y., Li J.-B., Zhang S.-L., Guo Q.-X., Liu L. (2009). Synthesis of Aromatic Esters via Pd-catalyzed Decarboxylative Coupling of Potassium Oxalate Monoesters with Aryl Bromides and Chlorides. J. Am. Chem. Soc..

[50] Jatoi A.H., Pawar G.G., Robert F., Landais Y. (2019). Visible-light Mediated Carbamoyl Radical Addition to Heteroarenes. Chem. Commun..

[51] Tlili A., Lakhdar S. (2021). Acridinium Salts and Cyanoarenes as Powerful Photocatalysts: Opportunities in Organic Synthesis. Angew. Chem. Int. Ed..

[52] Shang T.Y., Lu L.H., Cao Z., Liu Y., He W.M., Yu B. (2019). Recent advances of 1,2,3,5-tetrakis(carbazol-9-yl)-4,6-dicyanobenzene (4CzIPN) in photocatalytic transformations. Chem. Commun..

[53] Petersen W.F., Taylor R.J.K., Donald J.R. (2017). Photoredox-catalyzed Procedure for Carbamoyl Radical Generation: 3,4-Dihydroquinolin-2-one And Quinolin-2-one Synthesis. Org. Biomol. Chem..

[54] Wang P.-Z., Chen J.-R., Xiao W.-J. (2019). Hantzsch esters: An emerging versatile class of reagents in photoredox catalyzed organic synthesis. Org. Biomol. Chem..

[55] Cheng X., Huang W. (2016). Hantzsch Esters as Multifunctional Reagents in Visible-Light Photoredox Catalysis. Synlett.

[56] Alandini N., Buzzetti L., Favi G., Schulte T., Candish L., Collins K.D., Melchiorre P. (2020). Amide Synthesis by Nickel/Photoredox-Catalyzed Direct Carbamoylation of (Hetero)Aryl Bromides. Angew. Chem. Int. Ed..

[57] Cardinale L., Konev M.O., Jacobi von Wangelin A. (2020). Photoredox-Catalyzed Addition of Carbamoyl Radicals to Olefins: A 1,4-Dihydropyridine Approach. Chem. Eur. J..

[58] Matsuo B.T., Oliveira P.H.R., Correia J.T.M., Paixao M.W. (2021). Carbamoylation of Azomethine Imines via Visible-Light Photoredox Catalysis. Org. Lett..

[59] Kim I., Park S., Hong S. (2020). Functionalization of Pyridinium Derivatives with 1,4-Dihydropyridines Enabled by Photoinduced Charge Transfer. Org. Lett..

[60] Ryu I., Sonoda N. (1996). Free-Radical Carbonylations: Then and Now. Angew. Chem. Int. Ed..

[61] Ryu I., Sonoda N., Curran D.P. (1996). Tandem Radical Reactions of Carbon Monoxide, Isonitriles, and Other Reagent Equivalents of the Geminal Radical Acceptor/Radical Precursor Synthon. Chem. Rev..

[62] Chatgilialoglu C., Crich D., Komatsu M., Ryu I. (1999). Chemistry of Acyl Radicals. Chem. Rev..

[63] Sumino S., Fusano A., Fukuyama T., Ryu I. (2014). Carbonylation Reactions of Alkyl Iodides through the Interplay of Carbon Radicals and Pd Catalysts. Acc. Chem. Res..

[64] Peng J.-B., Geng H.-Q., Wu X.-F. (2019). The Chemistry of CO: Carbonylation. J. Chem..

[65] Brennfuhrer A., Neumann H., Beller M. (2009). Palladium-Catalyzed Carbonylation Reactions of Aryl Halides and Related Com-pounds. Angew. Chem. Int. Ed..

[66] Wu X.-F., Neumann H., Beller M. (2013). Synthesis of heterocycles via palladium-catalyzed carbonylations. Chem. Rev..

[67] Peng J.-B., Wu F.-P., Wu X.-F. (2019). First-Row Transition-Metal-Catalyzed Carbonylative Transformations of Carbon Electrophiles. Chem. Rev..

[68] Kawamoto T., Sato A., Ryu I. (2015). Photoinduced Aminocarbonylation of Aryl Iodides. Chem. Eur. J..

[69] Chow S.Y., Stevens M.Y., Akerbladh L., Bergman S., Odell L.R. (2016). Mild and Low-Pressure *fac*-Ir(ppy)_3_-Mediated Radical Ami-nocarbonylation of Unactivated Alkyl Iodides through Visible-Light Photoredox Catalysis. Chem. Eur. J..

[70] Zhao S., Mankad N.P. (2019). Metal-catalysed radical carbonylation reactions. Catal. Sci. Technol..

[71] Singh J., Sharma S., Sharma A. (2021). Photocatalytic Carbonylation Strategies: A Recent Trend in Organic Synthesis. J. Org. Chem..

[72] Lu B., Cheng Y., Chen L.-Y., Chen J.-R., Xiao W.-J. (2019). Photoinduced Copper-Catalyzed Radical Aminocarbonylation of Cycloketone Oxime Esters. ACS Catal..

[73] Forni J.A., Micic N., Connell T.U., Weragoda G., Polyzos A. (2020). Tandem Photoredox Catalysis: Enabling Carbonylative Amidation of Aryl and Alkylhalides. Angew. Chem. Int. Ed..

[74] Kwiatkowski M.R., Alexanian E.J. (2019). Transition-Metal (Pd, Ni, Mn)-Catalyzed C-C Bond Constructions Involving Unactivated Alkyl Halides and Fundamental Synthetic Building Blocks. Acc. Chem. Res..

[75] Torres G.M., Liu Y., Arndtsen B.A. (2020). A dual light-driven palladium catalyst: Breaking the barriers in carbonylation reactions. Science.

[76] Kathe P., Fleischer I. (2020). Light expands a catalyst‘s repertoire. Science.

[77] Sardana M., Bergman J., Ericsson C., Kingston L.P., Schou M., Dugave C., Audisio D., Elmore C.S. (2019). Visible-Light-Enabled Aminocarbonylation of Unactivated Alkyl Iodides with Stoichiometric Carbon Monoxide for Application on Late-Stage Carbon Isotope Labeling. J. Org. Chem..

[78] Cartier A., Levernier E., Dhimane A.L., Fukuyama T., Ollivier C., Ryu I., Fensterbank L. (2020). Synthesis of Aliphatic Amides through a Photoredox Catalyzed Radical Carbonylation Involving Organosilicates as Alkyl Radical Precursors. Adv. Synth. Catal..

[79] Cartier A., Levernier E., Corce V., Fukuyama T., Dhimane A.L., Ollivier C., Ryu I., Fensterbank L. (2019). Carbonylation of Alkyl Radicals Derived from Organosilicates through Visible-Light Photoredox Catalysis. Angew. Chem. Int. Ed..

[80] Veatch A.M., Alexanian E.J. (2020). Cobalt-catalyzed Aminocarbonylation of (Hetero)Aryl Halides Promoted by Visible Light. Chem. Sci..

[81] Debnath P. (2018). Recent Advances in the Synthesis of Amides via Oxime Rearrangements and its Applications. Curr. Org. Synth..

[82] Amin J.H., de Mayo P. (1963). The irradiation of aryl aldoximes. Tetrahedron Lett..

[83] Yadav L., Srivastava V., Yadav A. (2014). The Beckmann Rearrangement Executed by Visible-Light-Driven Generation of Vilsmeier–Haack Reagent. Synlett.

[84] Srivastava V., Singh P.P. (2017). Eosin Y catalysed photoredox synthesis: A review. RSC Adv..

[85] Tang L., Wang Z.-L., Wan H.-L., He Y.-H., Guan Z. (2020). Visible-Light-Induced Beckmann Rearrangement by Organic Photoredox Catalysis. Org. Lett..

[86] Nevesely T., Wienhold M., Molloy J.J., Gilmour R. (2021). Advances in the E → Z Isomerization of Alkenes Using Small Molecule Photocatalysts. Chem. Rev..

[87] Yuan P.-F., Huang T., He J., Huang X.-T., Jin X.-L., Sun C., Wu L.-Z., Liu Q. (2021). Controllable Z/E-selective synthesis of α-amino-ketoximes from N-nitrososulfonamides and aryl alkenes under neutral conditions. Org. Chem. Front..

[88] Zhang X., Rovis T. (2021). Photocatalyzed Triplet Sensitization of Oximes Using Visible Light Provides a Route to Nonclassical Beckmann Rearrangement Products. J. Am. Chem. Soc..

[89] Lowry M.S., Goldsmith J.I., Slinker J.D., Rohl R., Pascal R.A., Malliaras G.G., Bernhard S. (2005). Single-Layer Electroluminescent Devices and Photoinduced Hydrogen Production from an Ionic Iridium(III) Complex. Chem. Mater..

[90] Yu X.-Y., Zhao Q.-Q., Chen J., Xiao W.-J., Chen J.-R. (2020). When Light Meets Nitrogen-Centered Radicals: From Reagents to Catalysts. Acc. Chem. Res..

[91] Chen J.-R., Hu X.-Q., Lu L.-Q., Xiao W.-J. (2016). Visible Light Photoredox-Controlled Reactions of N-Radicals and Radical Ions. Chem. Soc. Rev..

[92] Zhao Y., Xia W. (2018). Recent Advances in Radical-Based C-N Bond Formation via Photo-/Electrochemistry. Chem. Soc. Rev..

[93] Lee W., Jeon H.J., Jung H., Kim D., Seo S., Chang S. (2021). Controlled Relay Process to Access N-Centered Radicals for Catalyst-free Amidation of Aldehydes under Visible Light. J. Chem..

[94] Jeon H.J., Lee W., Seo S., Chang S. (2021). N-Chloro-N-sodio-carbamates as a Practical Amidating Reagent for Scalable and Sustainable Amidation of Aldehydes under Visible Light. Org. Process Res. Dev..

[95] Liu M.-S., Shu W. (2020). Catalytic, Metal-Free Amide Synthesis from Aldehydes and Imines Enabled by a Dual-Catalyzed Umpolung Strategy under Redox-Neutral Conditions. ACS Catal..

[96] Ning Y., Wang S., Li M., Han J., Zhu C., Xie J. (2021). Site-specific Umpolung amidation of carboxylic acids via triplet synergistic catalysis. Nat. Commun..

[97] Brachet E., Ghosh T., Ghosh I., Konig B. (2015). Visible Light C-H Amidation of Heteroarenes with Benzoyl Azides. Chem. Sci..

[98] Ng C.-O., Feng H., Cheng S.-C., Xiao Y., Lo L.T.-L., Ko C.-C. (2018). Photoredox Catalysis of Cyclometalated Ir^III^ Complex for the Conversion of Amines to Fluorinated Alkyl Amides. Asian J. Org. Chem..

[99] Tian H., Shimakoshi H., Ono T., Hisaeda Y. (2019). Visible Light-Driven, One-pot Amide Synthesis Catalyzed by the B12 Model Complex under Aerobic Conditions. Chempluschem.

[100] Yadav A.K., Srivastava V.P., Yadav L.D.S. (2013). Visible-light-mediated eosin Y catalyzed aerobic desulfurization of thioamides into amides. New J. Chem..

[101] Zhou H., Lu P., Gu X., Li P. (2013). Visible-light-mediated nucleophilic addition of an alpha-aminoalkyl radical to isocyanate or isothiocyanate. Org. Lett..

[102] Zheng S., Primer D.N., Molander G.A. (2017). Nickel/Photoredox-Catalyzed Amidation via Alkylsilicates and Isocyanates. ACS Catal..

[103] Malacarne M., Protti S., Fagnoni M. (2017). A Visible-Light-Driven, Metal-free Route to Aromatic Amides via Radical Arylation of Isonitriles. Adv. Synth. Catal..

[104] Cannalire R., Amato J., Summa V., Novellino E., Tron G.C., Giustiniano M. (2020). Visible-Light Photocatalytic Functionalization of Isocyanides for the Synthesis of Secondary Amides and Ketene Aminals. J. Org. Chem..

[105] Yi M.-J., Zhang H.-X., Xiao T.-F., Zhang J.-H., Feng Z.-T., Wei L.-P., Xu G.-Q., Xu P.-F. (2021). Photoinduced Metal-Free α-C(sp^3^)–H Carbamoylation of Saturated Aza-Heterocycles via Rationally Designed Organic Photocatalyst. ACS Catal..

[106] Wu Y.-J., Liao G., Shi B.-F. (2022). Stereoselective construction of atropisomers featuring a C–N chiral axis. Green Synth. Catal..

[107] Cambié D., Bottecchia C., Straathof N.J.W., Hessel V., Noël T. (2016). Applications of Continuous-Flow Photochemistry in Organic Synthesis, Material Science, and Water Treatment. Chem. Rev..

[108] Peng J.-B., Liu X.-L., Li L., Wu X.-F. Palladium-catalyzed enantioselective carbonylation reactions. Sci. China Chem..

